# Parasites of Arctic char (*Salvelinus alpinus*) in North America: a systematic literature review and an analysis of contemporary data from anadromous populations from Nunavut

**DOI:** 10.1007/s11160-026-10068-x

**Published:** 2026-06-20

**Authors:** Les N. Harris, Phillip R. Morrison, Michael W. Johnson, Sara Bolduc, Ariane Côté, Amélie Papillon, Justine Lapointe, Xuehan Qu, David J. Yurkowski, Connor W. Faulkner, Colin P. Gallagher, Lauren N. Wiens, Matthew J. H. Gilbert, Jean-Sébastien Moore, Sandra A. Binning

**Affiliations:** 1 Arctic Fisheries and Marine Mammal Science Division, Fisheries and Oceans Canada, Winnipeg, MB Canada; 2 Department of Resource Management and Protection, Vancouver Island University, Nanaimo, BC Canada; 3 North/South Consultants Inc., Winnipeg, MB Canada; 4 Département de Biologie, Université Laval, Québec, QC Canada; 5 Département de Sciences Biologiques, Université de Montréal, Montreal, QC Canada; 6 Institut des Sciences de la Mer, Université de Québec À̇̇̇̇ Rimouski, Rimouski, QC Canada; 7 Institute of Arctic Biology and Department of Biology and Wildlife, University of Alaska Fairbanks, Fairbanks, AK USA; 8 Département de Biologie, Institut de Biologie Intégrative et des Systèmes, Université Laval, Quebec City, QC Canada; 9 Ressources Aquatiques Québec (RAQ), Rimouski, QC Canada

**Keywords:** Aquatic ecosystems, Baseline monitoring, Biodiversity, Climate change, Host-parasite interactions, Infection prevalence

## Abstract

Parasites play a critical yet understudied role in Arctic ecosystems, particularly within key subsistence species such as Arctic char (*Salvelinus alpinus*). As the Arctic undergoes rapid climate-driven change, establishing baseline parasite biodiversity is essential for monitoring ecological shifts and anticipating emerging health and conservation concerns. This study provides the most comprehensive assessment to date of Arctic char parasites in the North American Arctic by integrating a systematic literature review with new empirical data from 378 Arctic char collected across five Nunavut areas (2020–2024). The literature review (30 studies, 1956–2024) documented 67 parasite taxa—48 identified to species—with helminths (nematodes, cestodes, trematodes, acanthocephalans) dominant. Overall, 82% of taxa occurred in anadromous Arctic char and 54% in freshwater forms, including 22 species unique to anadromous fish. Our empirical assessment identified 35 parasite taxa—23 to species level—including several previously unreported from Arctic char in this region. Common and widespread parasites documented in the empirical assessment included *Brachyphallus crenatus*, *Derogenes varicus*, *Bothrimonus sturionis*, and *Alloopistholecithum gibbosum*, although notable spatial variation occurred in community composition and infection intensity. The occurrence of rare and previously unreported parasites may suggest emerging shifts in host–parasite dynamics potentially linked to changing prey availability and northward species range expansions. Together, these findings establish an essential baseline for Arctic char parasite biodiversity and highlight the need for continued monitoring to track future changes driven by climate warming, shifting food webs, and their implications for fish health and food security in northern Indigenous communities.

**Supplementary Information:**

The online version contains supplementary material available at 10.1007/s11160-026-10068-x.

## Introduction

Parasites are ubiquitous across both terrestrial and aquatic ecosystems, affecting a wide range of organisms from bacteria to vertebrates, with fishes hosting a particularly diverse array (Poulin and Morand 2000; Iwanowicz 2011; Timi and Poulin 2020). Infection with parasites can cause mechanical damage (e.g., gill lamellae fusion, tissue replacement), alter physiological states (e.g., cell proliferation, immune response, altered growth), and impair reproduction (e.g., resource diversion from gonadal development) in fish hosts, potentially leading to host death and population declines (Sindermann 1987; Iwanowicz 2011; Timi and Poulin 2020). Beyond individual effects, parasites also influence aquatic populations, communities, and ecosystems (Lafferty et al. 2008; Sures et al. 2017). The multi-scale impacts of parasites on fishes and aquatic ecosystems have economic consequences for aquaculture, commercial, subsistence, and recreational fishing, and hobbyist industries (Jansen et al. 2012; Krkošek et al. 2009; Krkošek 2017; Rao et al. 2013; Cooke et al. 2018). Some parasites, for example, anisakid nematodes, can also pose health risks to humans when raw or undercooked fish flesh is consumed (Rahmati et al. 2020). Despite advances in the collective understanding of host-specific and ecological roles of parasites in aquatic ecosystems, significant questions remain, particularly for Arctic fishes and Arctic aquatic ecosystems, where research is relatively scarce compared to more southern locations (Côté et al. 2021). This knowledge gap hinders our understanding of parasite impacts on fish communities and their contributions to regional biodiversity and ecosystem functioning (Timi and Poulin 2020) which is compounded by the fact that the Arctic is among the most rapidly changing regions on Earth.

The Arctic is warming at an alarming rate, with estimates indicating the region is warming four times faster than the global average (Rantanen et al. 2022). This phenomenon, known as Arctic amplification (Serreze and Barry 2011), is significantly affecting freshwater and marine ecosystems at high latitudes (Lehnherr et al. 2018; Yurkowski et al. 2018). Arctic amplification raises temperatures across aquatic habitats, alters river flows and affects sea ice dynamics including lengthening ice-free seasons (Reist et al. 2006; Koenigk et al. 2020). Warmer waters are also enabling northward expansion of sub-Arctic marine species with the potential to reshape Arctic ecosystems and host-parasite dynamics (Yurkowski et al. 2018; Falardeau et al. 2022; Sokolov et al. 2024). For example, forage fishes such as capelin (*Mallotus villosu*s) and sand lance (*Ammodytes* spp.) are expanding their range into previously unoccupied Arctic waters, altering food webs and trophic dynamics as they are key prey for many species (Falardeau et al. 2022; Ulrich and Tallman 2021). The influx of these species into the Arctic environments affects many levels of the marine food web, from primary producers (Kraft et al. 2013) to fishes (Campana et al. 2020; Falardeau et al. 2022) and marine mammals (Ferguson et al. 2010). These altered species interactions will undoubtedly have an impact on the emergence of additional parasites in Arctic marine habitats and/or influence the prevalence and transmission of endemic parasites (Hoberg et al. 2013) potentially impacting human health in Indigenous communities reliant on subsistence fishing and hunting (Brockington et al. 2023). Despite these growing concerns, the Arctic lacks long-term data needed to track parasite biodiversity and monitor shifts in host-parasite relationships driven by climate and other forms of anthropogenic change (Hoberg et al. 2013). Establishing baseline data is thus crucial for detecting emerging parasites, particularly in species such as Arctic char (*Salvelinus alpinus*) that are vital for subsistence, as food insecurity remains a pressing issue in the region (Panikkar and Lemmond 2020; Desautels et al. 2023).

Arctic char is an ecologically, culturally and economically important species throughout its Holarctic distribution, inhabiting both marine and freshwater environments (Johnson 1980; Taylor 2016; Weinstein et al. 2024). It displays diverse life history strategies, including anadromous, freshwater resident, and landlocked forms (Johnson 1980; Reist et al. 2013). Across Inuit Nunangat (Inuit homeland in Canada), Arctic char is essential to Inuit culture, economy, and food security, supporting subsistence harvesting in all communities (Priest and Usher 2004) and contributing to Indigenous-run commercial fisheries in several areas (Galappaththi et al. 2022). This species faces multiple threats from climate change and anthropogenic pressures, including increased shipping and industrial development throughout its geographic distribution. Its broad Holarctic distribution and use of both freshwater and marine habitats make it a valuable model for monitoring Arctic species responses to climate change (Lehnherr et al. 2018; Côté et al. 2021; Layton et al. 2021). Arctic char may serve as a sentinel species for monitoring ecosystem-level changes in Arctic aquatic habitats. For example, shifts in food webs (Falardeau et al. 2022; Faulkner et al. 2025) may lead to changes in parasite dynamics in both marine and freshwater systems (Marcogliese 2001, 2008). A clear understanding of contemporary parasite diversity in Arctic char is therefore needed to establish this species as a valuable monitoring target, support detection of future changes in Arctic food webs, and improve predictions of how host–parasite systems may respond to climate change at local and regional scales. This knowledge is also critical for assessing current impacts of parasitic infections on Arctic char performance, migrations, population dynamics, and overall health. However, limited data on Arctic char parasites, often restricted to a few locations, as well as a lack of a synthesis of past and recent literature on the topic means that only fragmented snapshots exist. This lack of comprehensive information makes it difficult to assess the current state of knowledge throughout the species range, hindering our ability to compare current and historical parasite records, which is vital for future monitoring and predicting ecological changes.

Due to the limited information on Arctic char parasites across their range and the need for baseline data to support monitoring, our objectives are two-fold. First, we synthesize available literature on the parasites infecting Arctic char in North America to assess parasite diversity, abundance and prevalence. Contemporary samples used in this study span a large area of the Canadian Arctic and, for comparative purposes of parasites found in the literature review and our empirical assessment, we limited our review to North America. Although some Indigenous communities in North America relying on Arctic char have reported recent increases in parasites in the fish they catch (e.g., Falardeau et al. 2022), no comprehensive scientific review has evaluated parasite diversity, abundance, and prevalence in the region. Second, we update this knowledge by analyzing recent Arctic char parasite data collected from multiple sites across Nunavut, Canada, including the Cambridge Bay, Naujaat, Rankin Inlet, Sanirajak and Sanikiluaq areas. This area encapsulates the majority of the Arctic distribution of Arctic char in North America. We compare parasite communities across these locations to highlight regional differences. While baseline data remain sparse in many areas, this review and empirical evaluation offer the most extensive assessment of Arctic char parasites in North America. Our findings identify parasites of concern for northern communities, explore potential climate change impacts, and highlight areas requiring further research.

## Materials and methods

### Literature review

To assess current knowledge of parasite prevalence, intensity, and abundance in Arctic char across North America, we used a combined approach. First, we conducted a systematic literature review using targeted keyword searches in Web of Science (WOS) and CAB Abstracts (CAB): (“Salvelinus alpinus” OR “Arctic Char”?) AND Parasit*, and (“Salvelinus alpinus” OR “Arctic Char”?) AND Infect*. The initial search (February 5, 2020) returned 473 articles in WOS and 486 in CAB. After removing duplicate records, 579 unique articles were screened by title and abstract to retain studies reporting Arctic char parasites from Arctic North America (Canada, Alaska, Greenland), including experimental, observational, and review studies from wild or farmed populations. Nineteen articles were reviewed in full, of which 13 reporting prevalence, intensity, and/or abundance were retained (Fig. S1). A second search (August 6, 2023; filtered from February 6, 2020) yielded 62 (WOS) and 38 (CAB) articles; after removing duplicate records from WOS, 67 unique articles were screened. Two articles were reviewed in full, with one meeting inclusion criteria (Fig. S1). A third search (April 23, 2025; filtered from August 7, 2023) yielded 15 (WOS) and 11 (CAB) articles; after removing duplicate records from WOS, 24 unique articles were screened, five reviewed in full, and one retained (Fig. S1).

Second, we screened reference lists of included studies using targeted keywords (e.g., char, parasite, and North American regions such as Québec), identifying 10 additional relevant studies. Third, we incorporated known grey literature (e.g., theses, government reports, unpublished data; Paez 2017) containing information on Arctic char parasites in North America.

Second, we performed a more targeted search of the literature by searching through the reference list of the selected articles for additional titles that seemed relevant for our search (i.e., keywords such as char(r), parasite, specific geographic regions known to be in North America [e.g., Québec]). From these relevant titles, we were able to identify a further 10 studies that met our search criteria, and were subsequently included in our study. Third, we included known gray literature (theses, government documents, unpublished data; Paez 2017) containing information on Arctic char parasites in North America. The goal of this combined approach was to ensure we compiled the most comprehensive dataset of Arctic char parasites in North America as possible rather than apply a rigorous and fully repeatable search process that knowingly missed key literature (details of our literature review process are highlighted in Fig. S1).

For gray literature, we followed the same procedure as described above, using ProQuest and Sofia databases to find theses, and the Federal Science Libraries Network (FSLN) to find government documents. This research yielded 19 documents with ProQuest, 33 with Sofia and 21 with FSLN. A total of 51 documents were kept after deleting duplicates. Following title and abstract screening, 24 articles were retained to be read in full. Of these, eight had data on parasite prevalence, intensity and/or abundance and were included in the study. We removed three papers that sampled sites in Alaska west of the Mackenzie River due to the mis-identification of Dolly Varden (*Salvelinus malma*) as Arctic char in these areas prior to the 1980’s (Weinstein et al. 2024). From the remaining 30 articles we manually extracted information on the geographic location studied (i.e. GPS coordinates), parasite species names, average parasite prevalence, intensity and abundance for each species (see below for details on how these metrics were calculated). Details of the articles included and how they were identified are included in Table S1**.**

### Sample collection for contemporary parasite data from Nunavut

**Fig. 1:**
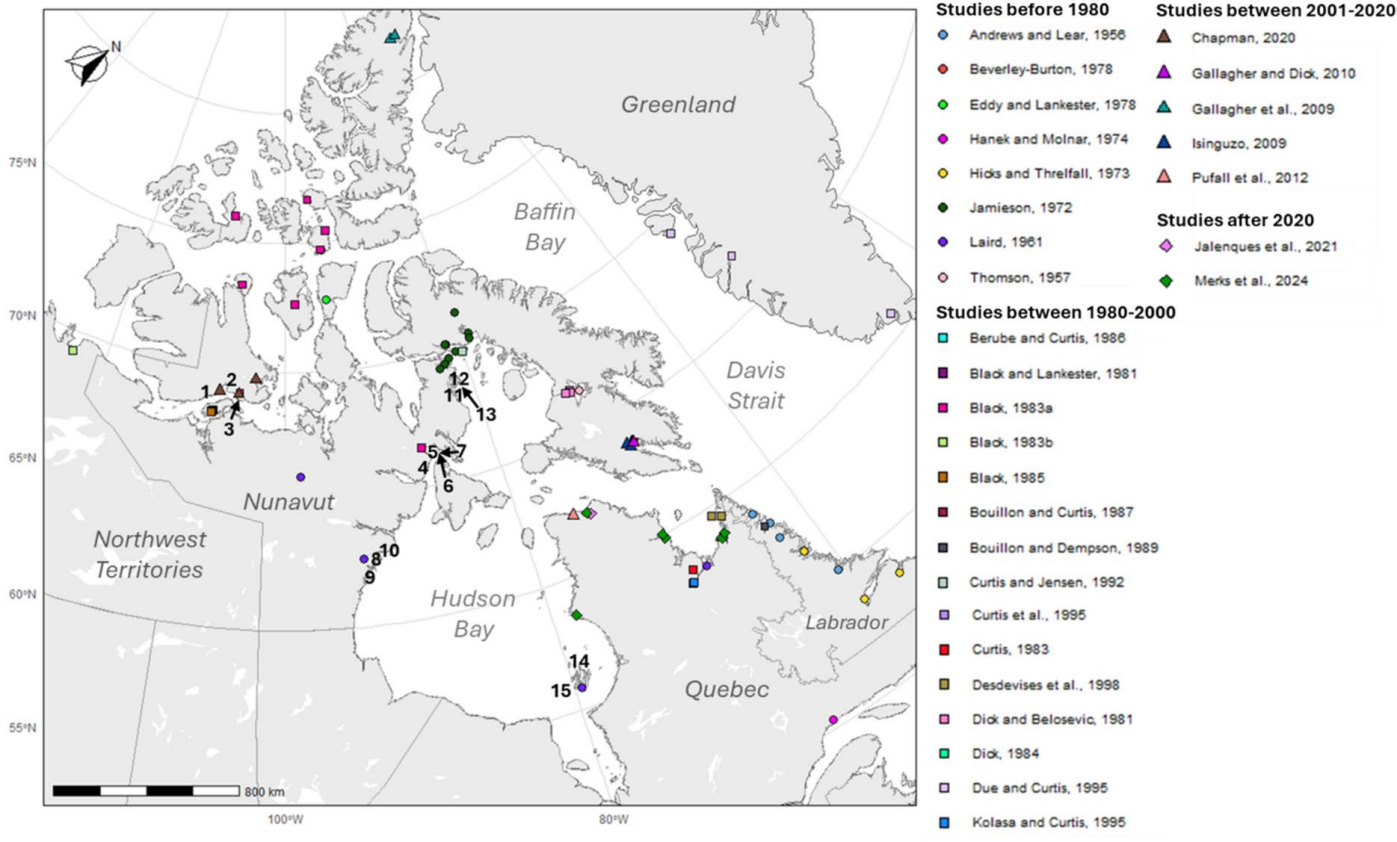
Geographic distribution of study sites included in the Arctic char (*Salvelinus alpinus*) parasite literature review, showing locations of all published investigations conducted from 1956 to 2024. Each symbol represents an individual study, with shapes denoting time periods (before 1980; 1980–2000; 2000–2020; after 2020) and colours corresponding to unique publications within each period. Numbers indicate contemporary sampling locations (2020‒2024) for the empirical assessment of parasites (see Table 1). Cambridge Bay area = 1–3, Naujaat area = 4–7, Rankin Inlet area = 8–10, Sanirajak area = 11–13 and Sanikiluaq area = 14–15

Samples for the empirical assessment of anadromous Arctic char parasites were collected from several areas in Arctic Canada spanning marine, estuarine and freshwater habitats from 2020 to 2024 (Fig. 1**, **Table 1). Anadromy was inferred based on sampling location and population identity, with fish captured in marine and estuarine environments, as well as those collected from freshwater lakes known to support anadromous populations, were presumed to be anadromous. These samples were collected as part of several past and current Fisheries and Oceans Canada (DFO) research initiatives in Nunavut. These included DFO stock assessment data-collection activities and varying community-based sampling programs. Samples were collected from five general areas in Nunavut that included 15 discrete sampling locations: Cambridge Bay (Byron Bay, Ekalluk, Gravel Pit), Naujaat (Kuugarjuk, Naujaat, North Pole River, Sipujaqtuu), Rankin Inlet (Akaalik, Corbett, Diana River), Sanirajak (Hall Beach, Hall Lake, Sanirajak) and Sanikiluaq (Tasirjuaq, Inutsualuit, Table 1). Depending on the research initiative or community-based sampling program, stomachs and/or entire viscera, full bodies or heads were collected, placed in plastic bags and frozen until parasite analysis. As such, the organs examined for parasites varied among regions, however complete digestive tracts were assessed in every individual. Biological data (length, weight, sex and maturity) were also opportunistically collected depending on the research initiative.

**Table 1:** Sampling locations for anadromous Arctic char collections used in the empirical assessment of parasites

Area	Sampling location	Habitat	Lat	Lon	Sample size	Year(s) collected
Cambridge Bay	Byron Bay	Estuary	68.9439	− 108.5353	30	2021
	Ekalluk	Estuary	69.0934	− 105.2956	41	2021
	Gravel Pit	Marine	69.0934	− 105.2956	58	2022
Naujaat	Kuugarjuk	Marine	66.4704	− 85.3099	29	2020
	Naujaat	Marine	66.5068	− 86.2237	31	2020
	North Pole River	Estuary	66.5305	− 86.7307	32	2020
	Sipujaqtuu	Estuary	66.3884	− 86.7194	23	2020
Rankin Inlet	Diana River	Estuary	62.8158	− 92.3402	2	2020
	Corbett Inlet	Marine	62.5146	− 92.4913	30	2020
	Akaalik	Marine	62.8270	− 91.3610	30	2020
Sanirajak	Hall Beach	Marine	68.7979	− 81.2371	9	2021
	Sanirajak	Marine	68.8420	− 81.6159	4	2021
	Hall Lake	Freshwater	68.6689	− 82.2637	25	2021
Sanikiluaq	Tasirjuaq	Marine	55.8350	− 79.8681	2	2024
	Inutsualuit	Freshwater	56.4051	− 79.1664	32	2024

Maps code refer to those in Fig. 1.Also shown is the habitat in which Arctic char were sampled, the sampling location coordinates (provided in decimal degrees), sample size and years of sample collection

### Parasite data collection

Each sample bag containing the viscera was removed from the freezer and allowed to thaw until organs could be separated while still remaining partially frozen. All organs present in each sample were separated into individual petri dishes. Fish sex and maturity were assessed when possible and gonad and liver weights (± 0.001 g) were recorded. For samples that included heads, the mouth and branchial cavity were examined for any attached parasites and cysts. Organs with a lumen (e.g., eyes, gastrointestinal tract, gallbladder, swim bladder etc.) were opened and contents were removed with a blunt probe or small steel spatula, under a dissecting microscope. Contents and organ linings were inspected for parasites and embedded cysts. Following a surface scan, small incisions with a sharp scalpel blade were used to check the interior of the heart, liver and spleen for encysted parasites.

All parasites were identified to the lowest taxonomic level possible using relevant keys, enumerated, and preserved in either 70% ethanol (long-term storage) or 95% DNA-grade ethanol. Digital camera-equipped microscopes were used to create photographic libraries of each parasite taxon to assist with identifications. Parasites that could not immediately be identified to species during necropsy were cleared, stained to highlight key internal organs (using standard taxon-specific techniques), and mounted on slides for more precise identification. Trematodes, cestodes, and acanthocephalans were stained for approximately 30 min, or until internal organs had absorbed sufficient colour, in a 70% ethanol-based carmine stain. Parasites were then removed from stain and placed briefly in an acid alcohol bath to remove excessive stain from the body wall, while leaving the organs well-stained. The parasites were then flushed in 70% ethanol to remove all acid and to stop the de-staining process. The specimens were then gradually transitioned, through serial stages, from 70 to 100% ethanol and cleared with methyl salicylate (to improve light transmission through tegument and visibility of the stained organs) before mounting on slides with Canada balsam. Nematoda were not stained but were cleared in 5% glycerine-alcohol to view internal features. Parasitic copepods did not require additional processing and metacercariae were identified using a combination of species-specific morphological features and infection site.

### Data analysis

Descriptive and statistical analyses were performed using the freely available R-based software Quantitative Parasitology on the Web (QPweb; version 1.0.15) (Reiczigel et al. 2019). Ecological terminology conforms to the definitions of Bush et al. (1997), and the quantification of parasites follows the recommendations of Rózsa et al. (2000). Not all organs or tissues were sampled in every Arctic char; therefore, to assess the parasite communities of the different host populations, hosts were included in the analyses if their body cavity and digestive tract (stomach, foregut, and hindgut, N = 378) were examined. Some organs such as the heart, swim bladder, and gills were not examined in all hosts.

For each parasite taxon, prevalence of infection (number of infected hosts/number of examined hosts) and mean intensity of infection (total number of parasites in a sample/total number of infected hosts) were calculated across all samples and by sampling area (i.e., locations within a sampling area combined, see Table 1). A 95% confidence interval (CI) was calculated using Sterne’s method (Reiczigel 2003) for prevalence, and by applying bias-corrected and accelerated bootstrap (*BC*_*a*_) CIs for mean intensity (Rózsa et al. 2000). Comparisons of parasite prevalence among sampling locations were done using Fisher’s Exact test, and mean intensity was compared by bootstrap analysis of variance (ANOVA, when number of sampling areas and number of hosts per sampling area permitted). Since parasites are often aggregated in their hosts, parasite aggregation was quantified by calculating Poulin’s index of discrepancy (*D*) (Poulin 1993). Values of *D* range between zero and one, with zero indicating an even distribution of parasites (low aggregation) across hosts, and one indicating that all parasites are in a single host (high/extreme aggregation) (Poulin 1993). The following community descriptors were also calculated for each sampling area: 1) number of hosts examined, 2) the total number of parasite individuals, 3) the component community species richness (the total number of different parasite species detected across all fish sampled within an area), 4) the mean infracommunity species richness (the average number of parasite species found within a single fish per area), ± standard deviation), 5) total prevalence, and 6) the total mean intensity (± *BC*_*a*_ 95% CIs).

To visualize patterns in parasite community composition across sampling locations, we first used non-metric dimensional scaling (NMDS), a non-parametric ordination technique that relies on the rank order of pairwise dissimilarities among groups while avoiding distributional assumptions of the data (Clarke and Warwick 2001). Area similarity (based on the Bray–Curtis dissimilarity index) in parasite composition was visualized with an NMDS plot using the *metaMDS* function in the *vegan* package (Oksanen et al. 2022) in R version 4.1.1 (R Core Team 2025). For this analysis, we included the parasites that could be identified to species level (N = 23) and an additional seven parasites that could only be identified to genus (N = 7), from hosts for which the full digestive tract was examined. To test for differences in parasite community composition among sampling regions, we treated the multivariate parasite matrix (Hellinger-transformed abundances for all parasite taxa) as the response, and Area as the predictor (grouping) variable in both the one-way multivariate analysis of variance (MANOVA) and the permutational multivariate analysis of variance (PERMANOVA; Anderson 2001). This allowed us to formally evaluate whether overall parasite community structure differed significantly among areas. Data visualizations were created with the *tidyverse* and *ggplot2* packages (Wickham et al. 2019). The NMDS analysis was conducted on a Hellinger-transformed distance matrix of parasite count data. The goodness of fit for the NMDS ordination was assessed using the stress value, where values < 0.05 indicate an excellent fit, < 0.1 indicate a good fit, and values < 0.2 represent a moderate fit that may warrant cautious interpretation (Clarke 1993).

Next, we generated a heatmap based on Bray–Curtis similarity indices. Parasite abundance data were first aggregated by location, summing counts of all parasite taxa for each unique site. Bray–Curtis dissimilarity values were then calculated using the vegdist() function from the VEGAN package, and then converted to similarity scores by subtracting each value from 1. The resulting similarity matrix was visualized as a clustered heatmap using the ComplexHeatmap package (Gu et al. 2016). Rows and columns represented sampling locations, and hierarchical clustering was applied to both axes to identify patterns of compositional similarity among sites.

### Historical and empirical species overlap

To minimize ambiguity associated with mixed taxonomic resolution and to enable a conservative comparison of parasite assemblages through time, only parasite records identified to species level were retained for comparative analyses. Genus-level (spp.) and higher-rank identifications were excluded. Species names from the historical literature review and the empirical assessment were standardized to account for recognized taxonomic updates and synonymous nomenclature before comparing taxa unique to each dataset and taxa shared between datasets. Overlap between datasets was visualized in R using the VennDiagram package (Chen and Boutros 2011).

## Results

### Systematic literature review

**Fig. 2:**
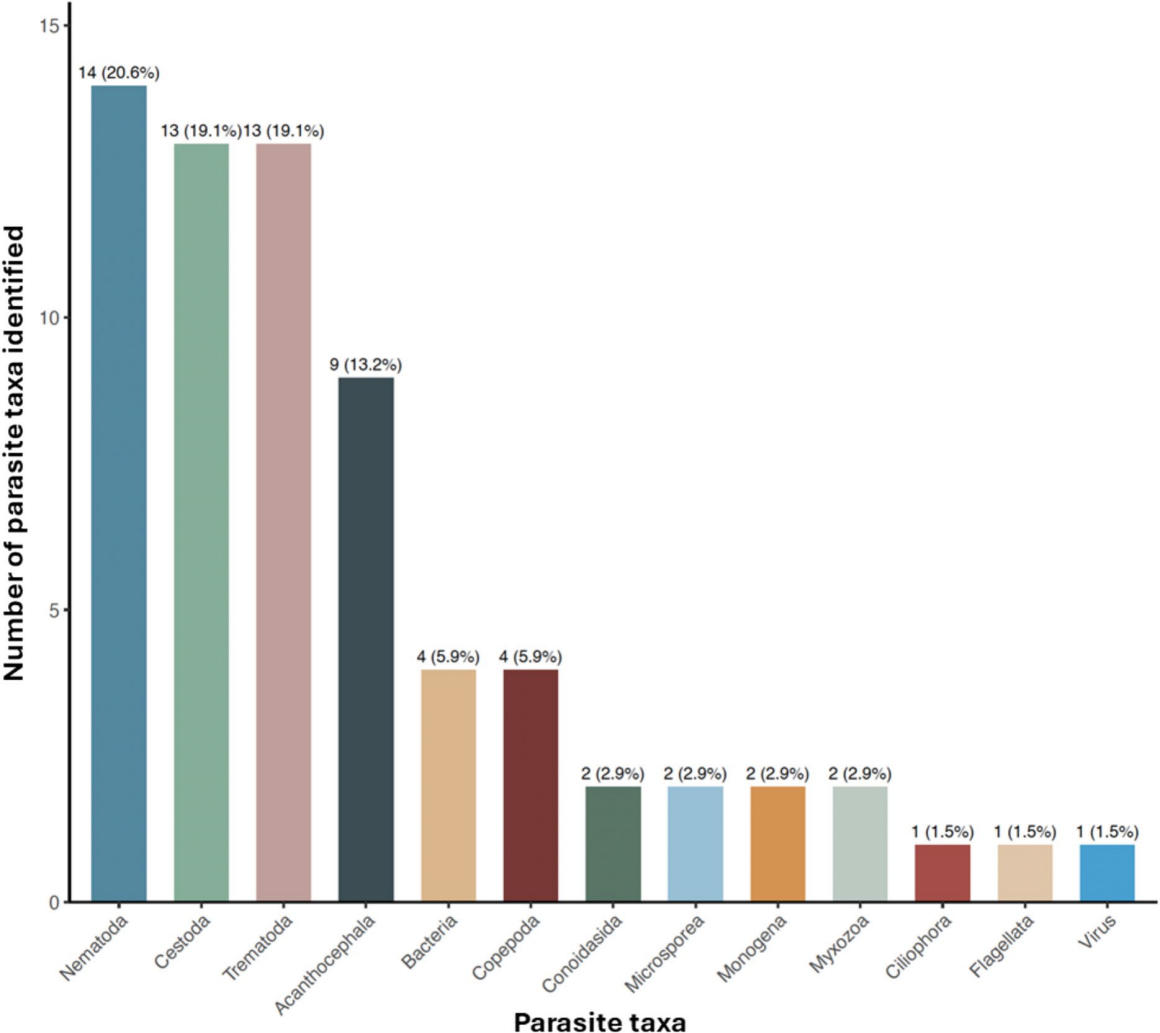
Bar plot of the number of parasite species (also including viruses and bacteria) identified within each broader parasite taxonomic group in the 30 historical studies of Arctic char from North American waters identified in the systematic literature review

Our literature review identified 30 studies with data on the abundance and/or prevalence of Arctic char parasites in Arctic North America (Table S1, Table S2). These studies were published between 1956 and 2024, with 24 studies (77%) published before 2000 and 8 studies (26%) before 1980 (Fig. S2). From these studies, 67 parasite taxa were identified, including 48 parasites identified to species level (Table S2). The parasite classes with the highest numbers of identified parasites are all helminths, 14 studies for (20.6%) nematodes, 13 (19.1%) for cestodes and trematodes, and nine for (13.2%) acanthocephalans (Fig. 2).

**Fig. 3:**
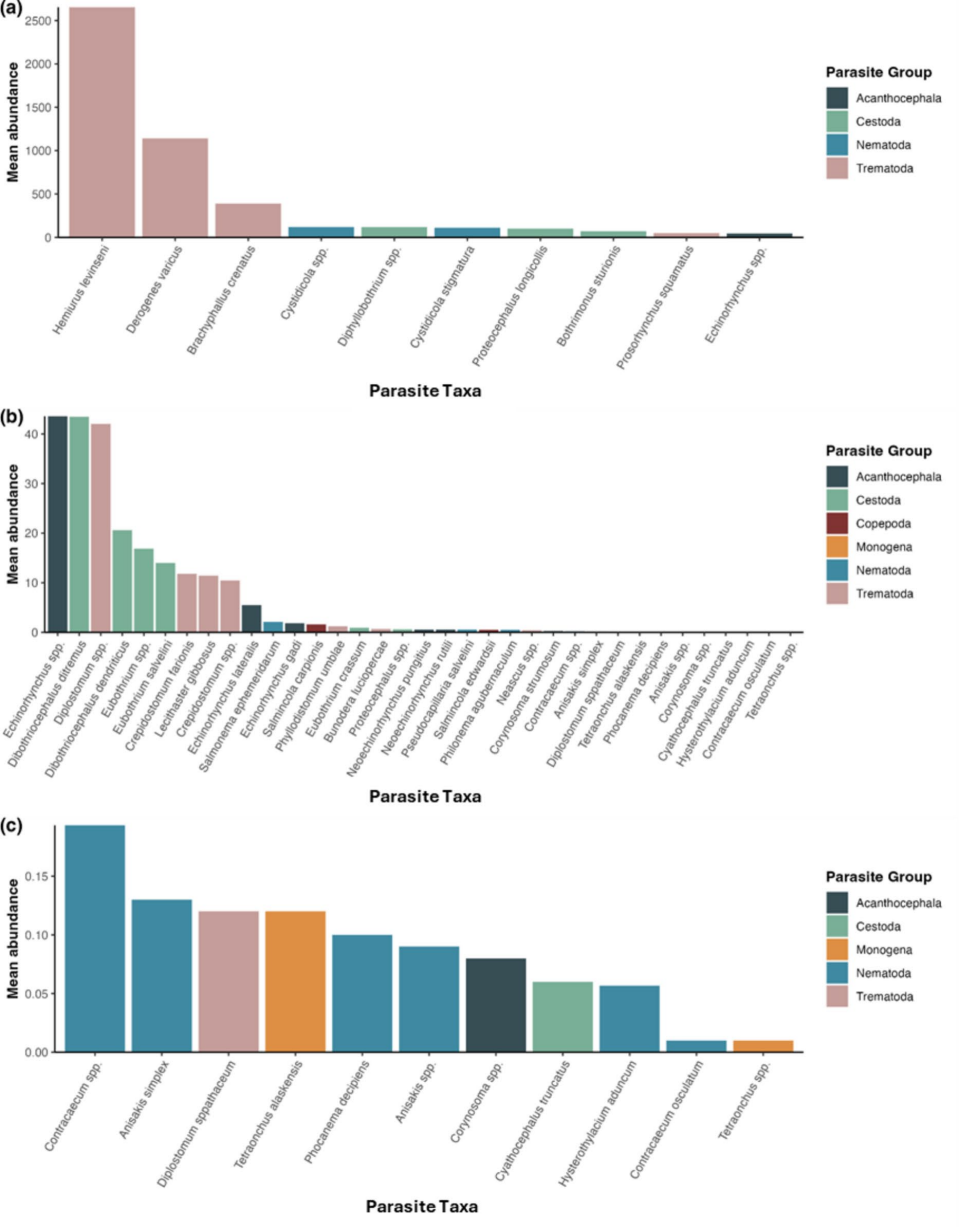
Histograms of the mean abundance of parasite taxa identified in the systematic literature review. **a** Average abundance of the ten most abundant parasites. **b** Average abundance of the other parasites, with *Echinorhynchus* spp. kept as a reference. **c** Average abundance of the ten least abundant parasites, with *Contracaecum* spp. kept as a reference. Twenty-four parasite taxa had no abundance data

Of all of the parasite taxa identified in the literature, 56 (82%) were found in anadromous Arctic char while 37 (54%) were found in freshwater Arctic char: 22 parasite species were identified exclusively in anadromous fish, three inhabiting exclusively freshwater, and eight in those of unknown life history (Table S2). The most prevalent parasite, present in 14 (45%) studies, was *Eubothrium salvelini* (Cestoda), followed by *Dibothriocephalus (syn. Diphyllobothrium*) spp. (Cestoda) present in 10 (32%) studies (Table S2). In the literature review studies, average prevalence was estimated for 98% of the identified parasite species, the average intensity for 67% and the average abundance for 68% (Table S2). Means of these statistics for each parasite taxa are reported in Table S2. The parasite with the highest mean prevalence (100%), mean intensity (2653) and mean abundance (2653) is *Hemiurus levinseni* (Trematoda, Fig. 3, Table S2, Figure S3). *Derogenes varicus* (Trematoda) had the second highest mean intensity (1143) and mean abundance (1141), followed by *Brachyphallus crenatus* (Trematoda) (mean intensity: 223, mean abundance: 389, Fig. 3, Table S2, Figure S3). The parasite taxon with the lowest mean prevalence (0.19%) is *Pomphorhynchus* spp. and the one with the lowest mean intensity (0.14) is *Phocanema decipiens* (Nematoda). *Contracaecum osculatum* (Nematoda) and *Tetraonchus* spp. (Monogena) are the ones with the lowest mean abundance (0.01, Fig. 3, Table S2, Figure S3).

### Contemporary parasite assessment

We assessed parasites in 378 contemporary anadromous Arctic char samples collected across five areas of Nunavut (Cambridge Bay, Naujaat, Rankin Inlet, Sanirjajak and Sanikiluaq) including 15 discrete sampling locations (Fig. 1). Among these, a total of 1639 organs were assessed including, fat/mesentery, gall bladder, gills, gonads, heart, liver, spleen, swim bladder, hindgut, foregut, and stomach. All sampled hosts were infected with at least one parasite species and a total of 392,619 parasites were counted across all samples. Of the 35 parasite taxa identified, thirty-four were identified at least to genus, 23 of which were identified to species (Table 2). Parasites were found in the body cavity or digestive tract of the combined host populations, and an additional six parasite taxa were found in either the heart, swim bladder, or on the gills. Among areas, the total number of parasites counted ranged from n = 16,928 (Sanikiluaq) to n = 136,799 (Sanirajak, Table 3). Total prevalence was 100% among all areas and total mean intensity ranged from 480 (Rankin Inlet, Table 3) to 3600 (Sanirajak, Table 3). Many of the parasites were not equally distributed among the host populations. Among the host population, parasite species richness ranged from 16 (Rankin Inlet) to 25 (Cambridge Bay) and mean infracommunity richness ranged from 4.8 (Rankin Inlet (± 1.5), Naujaat (± 2.2) and Sanirajak (± 2.2)) to 6.1 ± 1.7 (Cambridge Bay, Table 3). The mean infracommunity richness across all sites was 5.4 (± 2.0, Table 3).

**Table 2:** Summary of parasite taxa identified in Arctic char across all samples, showing the number of parasites, prevalence (%; with 95% confidence intervals), and mean (with 95% confidence intervals), median, and maximum intensity for each taxon

Group	Parasite	Number parasites	Prevalence (%)	Mean intensity	Median intensity	Max intensity	Areas noted
**Trematoda—Digenea**	*Brachyphallus crenatus*	215,050	83.6 (79.5–87.1)	681.0 (523.0–970.0)	161	23,040	CB, NA, RI, SJ, SQ
	*Hemiurus levinseni*	1612	9.0 (6.4–12.4)	47.4 (19.9–145.0)	13	829	RI, NA
	*Derogenes varicus*	59,243	75.9 (71.3–80.0)	206.0 (145.0–346.0)	26	9946	CB, NA, RI, SJ, SQ
	*Ichthyocotylurus* spp.*	464	26.3 (14.0–42.1)	46.4 (14.6–104.0)	16	199	CB
	Ichthyocotylurus erraticus*	10	66.7 (27.1–93.7)	2.5 (1.0–4.0)	2	5	SQ
	***Podocotyle angulata***	34	1.1 (0.4–2.7)	8.5 (1.5–19.2)	5	23	SJ, SQ
	*Podocotyle sinusacca*	1	0.3 (0.0–1.5)	1.0 (0.0–0.0)	1	1	CB
	*Alloopistholecithum gibbosum*	34,966	52.1 (46.9–57.2)	177.0 (113.0–304.0)	11	5280	CB, NA, RI, SJ, SQ
	*Prosorhynchus squamatus*	2367	6.6 (4.4–9.6)	94.7 (12.6–486.0)	3	2000	CB, NA, RI, SJ, SQ
	*Crepidostomum farionis*	833	9.8 (7.1–13.2)	22.5 (11.4–46.2)	4	237	CB, NA, RI, SJ, SQ
	*Crepidostomum* spp.	119	1.6 (0.7–3.4)	19.8 (2.0–54.5)	4	105	CB, NA
	***Steringotrema* spp.**	2	0.5 (0.1–1.9)	1.0 (0.0–0.0)	1	1	RI, SQ
**Cestoda**	***Bothriocephalus* spp.**	2	0.3 (0.0–1.5)	2.0 (0.0–0.0)	2	2	SQ
	*Bothrimonus sturionis*	53,466	68.0 (63.1–72.6)	208.0 (161.0–280.0)	27	3174	CB, NA, RI, SJ, SQ
	*Dibothriocephalus* spp.	731	24.1 (20.0–28.7)	8.0 (5.8–11.5)	2	74	CB, NA, SJ, SQ
	*Eubothrium salvelini*	2728	54.8 (49.6–59.8)	13.2 (9.8–19.5)	3	296	CB, NA, RI, SJ, SQ
	*Proteocephalus* spp.	6263	4.5 (2.7–7.1)	368.0 (12.9–1930.0)	3	5952	CB, SJ, SQ
**Nematoda**	*Pseudocapillaria (Ichthyocapillaria) salvelini*	41	0.8 (0.2–2.3)	13.7 (3.0–23.3)	6	32	SQ
	***Raphidascaris (Raphidascaris) acus***	388	2.4 (1.2–4.5)	43.1 (14.2–126.0)	15	249	SQ
	*Hysterothylacium aduncum*	179	16.1 (12.7–20.2)	2.9 (2.1–4.8)	2	28	CB, RI, NA, SQ
	*Hysterothylacium* spp.	37	0.8 (0.2–2.3)	12.3 (2.0–21.7)	5	30	CB, SJ
	*Salmonema ephemeridarum*	9	1.6 (0.7–3.4)	1.5 (1.0–2.0)	1	4	RI, NA
	*Philonema* spp.	6	1.3 (0.5–3.1)	1.2 (1.0–1.4)	1	2	CB, SJ
	*Anisakinae*	51	4.0 (2.3–6.4)	3.4 (2.0–6.5)	2	15	CB, NA, SJ
	Cystidicola farionis*	9020	32.6 (26.0–40.0)	158.0 (79.3–326.0)	6	2249	CB, NA, RI, SJ
	*Ascarophis* spp.*	11	1.4 (0.5–3.7)	2.8 (1.0–4.0)	2.5	5	NA
**Acanthocephala**	*Corynosoma* spp.	176	18.0 (14.4–22.2)	2.6 (1.9–4.3)	1	32	CB, RI, NA, SQ
	*Corynosoma wegeneri*	5	1.1 (0.4–2.7)	1.3 (1.0–1.5)	1	2	CB, SQ
	*Echinorhynchus gadi*	3915	52.9 (47.7–57.9)	19.6 (13.3–45.7)	4	1070	CB, NA, RI, SJ
	*Echinorhynchus leidyi*	695	8.7 (6.2–12.0)	21.1 (9.0–51.4)	4	271	SJ, SQ
	*Echinorhynchus salmonis*	26	1.6 (0.7–3.4)	4.3 (1.7–9.7)	2.5	14	CB
	*Neoechinorhynchus (Neoechinorhynchus) tumidus*	2	0.5 (0.1–1.9)	1.0 (0.0–0.0)	1	1	CB, SQ
**Copepoda**	Salmincola carpionis*	105	20.0 (9.4–34.9)	13.1 (2.1–44.5)	2	82	CB
	Salmincola edwardsii*	50	21.7 (14.2–31.5)	2.5 (1.7–3.5)	1.5	7	CB, RI
	*Salmincola* spp.*	12	7.7 (2.7–18.1)	3.0 (1.0–5.0)	1	9	RI

Data are based on 378 fish collected from five areas in Nunavut (CB = Cambridge Bay, NA = Naujaat, RI = Rankin Inlet, SJ = Sanirajak, SQ = Sanikiluaq). Note that not all organs were examined in every fish; in total, 1,639 organs were assessed across all individuals. Asterisks (*) indicate parasites detected in organs (e.g., heart, gills) that were not examined in all samples and taxa in bold represent species previously not reported in Arctic char, at least in Canada

**Table 3:** Summary of parasite infracommunity metrics for Arctic char (*Salvelinus alpinus*) sampled across five areas of Nunavut (Cambridge Bay, Rankin Inlet, Naujaat, Sanirajak, and Sanikiluaq) between 2020 and 2024

Host population	Cambridge Bay	Rankin Inlet	Naujaat	Sanirajak	Sanikiluaq	Combined
Number of examined hosts	129	62	115	38	34	378
Total number of parasites	104,743	29,784	104,365	136,799	16,928	392,619
Parasite species richness	25	16	17	16	20	35
Mean infracommunity richness ± S.D	6.1 ± 1.7	4.8 ± 1.5	4.8 ± 2.2	4.8 ± 2.2	5.7 ± 2.5	5.4 ± 2.0
Total prevalence	100	100	100	100	100	100
Total mean intensity (95% CI)	813 (648–978)	480 (350–611)	909 (609–1,208)	3600 (1,300–5,900)	498 (135–861)	1039 (777–1302)

Values shown include the number of examined hosts, total number of parasites, parasite species richness, mean infracommunity richness (± S.D.), overall prevalence (% of infected hosts), and mean intensity of infection (± 95% CI). Combined values are presented across all sampling locations

**Fig. 4:**
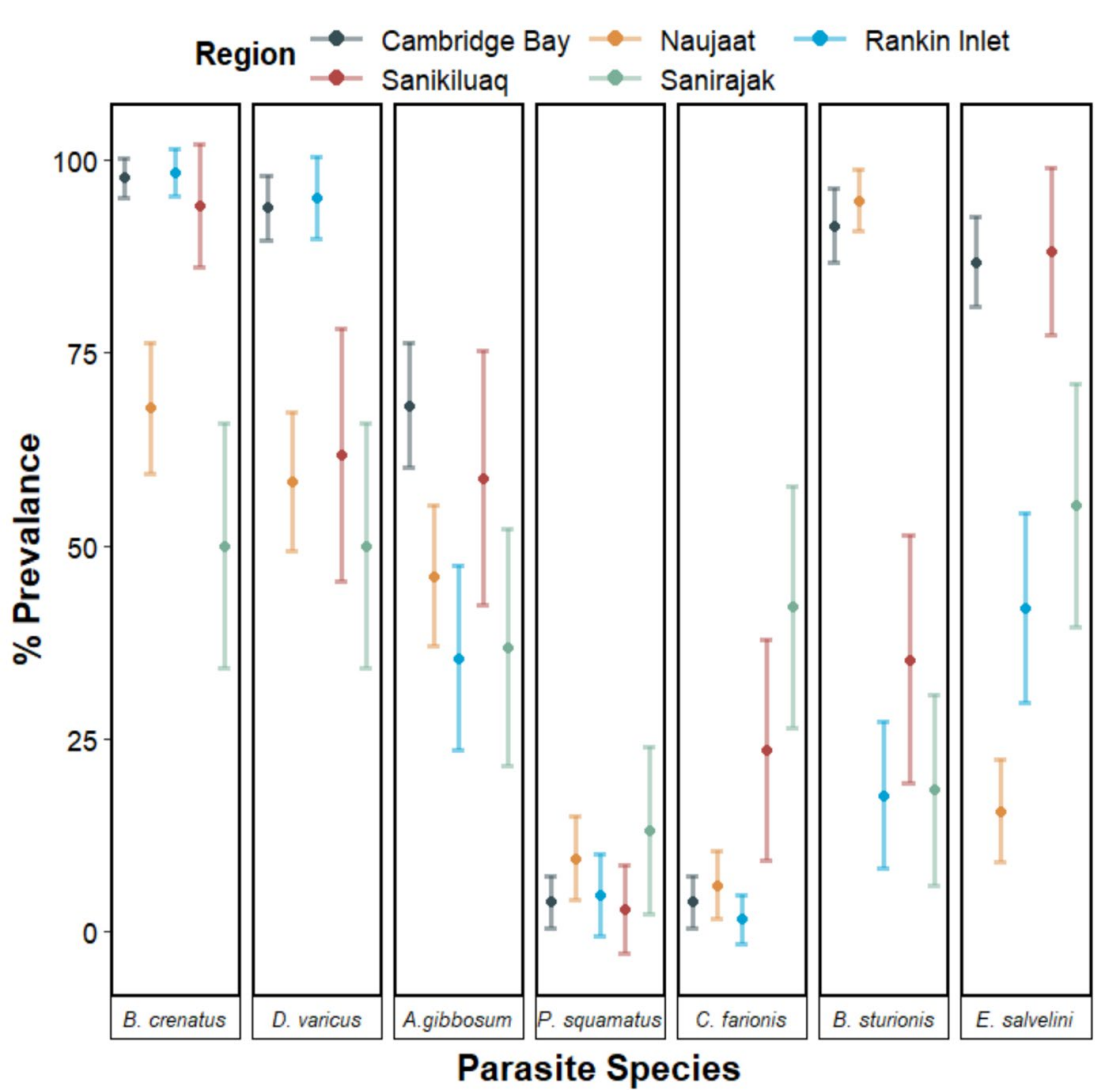
Prevalence (% of infected hosts, with 95% bootstrapped confidence intervals) of the seven parasite taxa detected in all five sampling areas of Nunavut (Cambridge Bay, Rankin Inlet, Naujaat, Sanirajak, and Sanikiluaq). These widespread species include the digeneans *Brachyphallus crenatus*, *Derogenes varicus, Alloopistholecithum gibbosum* (*syn. Lecithaster gibbosus), Prosorhynchus squamatus* and *Crepidostomum farionis*, and the cestodes *Bothrimonus sturionis* and *Eubothrium salvelini*

The most prevalent parasites across all areas were the trematodes *Brachyphallus crenatus* (316/378 hosts infected, 83.6%) and *Derogenes varicus* (287/378 hosts infected, 75.9%), the cestodes *Bothrimonus sturionis* (257/378 hosts infected, 68.0%) and *Eubothrium salvelini* (207/378 hosts infected, 54.8%), the acanthocephalan *Echinorhynus gadi* (200/378 hosts infected, 52.9%) and the trematode *Alloopistholecithum gibbosum* (*syn. Lecithaster gibbosus*, 197/378 hosts infected, 52.1%, Table 2). All other parasites had a prevalence of < 35%. These species, with the exception of *E. gadi* were also found in all sampling areas. *Ichthyocotylurus erraticus* had a mean prevalence of 66.7% however, only six hosts were inspected. Additional species found in all sampling areas included *Prosorhynchus squamatus* and *Crepidostomum farionis*. All the common species noted above, with the exception of *P. squamatus*, differed significantly (P < 0.01) in prevalence among sampling areas (Table S3, Fig. 4).

**Fig. 5:**
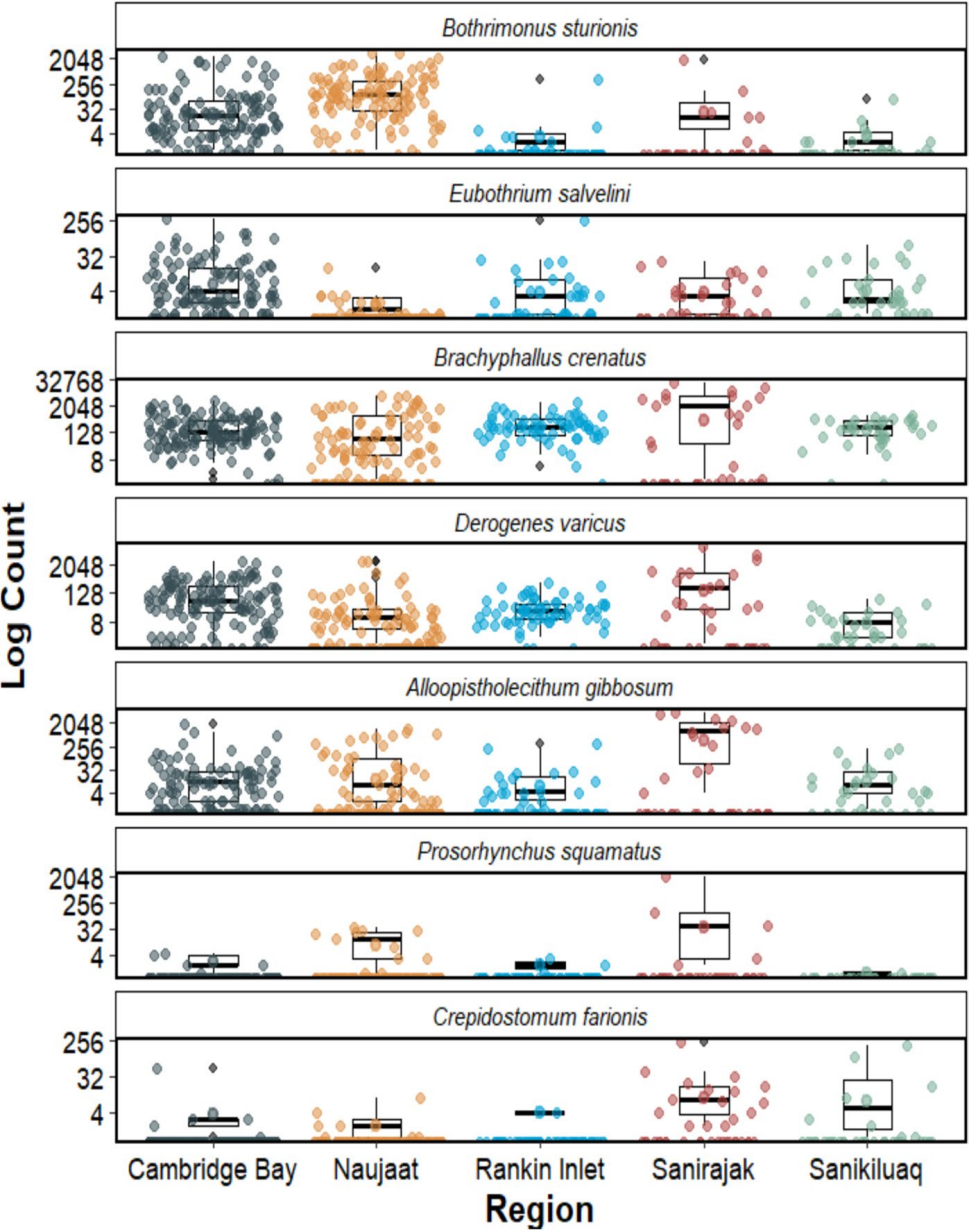
Intensity (total number of parasites in a sample/total number of infected hosts) of the seven parasite taxa detected in all five sampling regions of Nunavut (Cambridge Bay, Rankin Inlet, Naujaat, Sanirajak, and Sanikiluaq). These widespread species include the digeneans *Brachyphallus crenatus*, *Derogenes varicus, Alloopistholecithum gibbosum* (*syn. Lecithaster gibbosus), Prosorhynchus squamatus* and *Crepidostomum farionis*, and the cestodes *Bothrimonus sturionis* and *Eubothrium salvelini*. Boxplots show median (line), interquartile range (box), and values within 1.5 × IQR (whiskers), with individual observations overlaid

Similarly, the most abundant parasites were *B. crenatus* (215,050 total parasites; mean abundance = 568.9), *D. varicus* (59,243 total parasites; mean abundance = 156.7), *B. sturionis* (53,466 total parasites; mean abundance = 141.4), and *A. gibbosum* (34,966 total parasites; mean abundance = 92.5, Table 2). All other parasite taxa had a mean abundance of < 25. *B. crenatus* had the highest mean intensity of infection (681.0) followed by *Proteocephalus* spp. (368.0), *B. sturionis* (208.0), *D. varicus* (206.0), *A. gibbosum* (177.0, Table 2) and *Cystidicola farionis* (158.0). All other parasite taxa exhibited a mean intensity < 100. Parasite mean intensity of *B. crenatus*, *D. varicus* and *A. gibbosum* differed significantly among sampling areas (Table S4, Fig. 5).

Overall, most parasite taxa were strongly to extremely aggregated within hosts across regions, with many dispersion indices (D) ≥ 0.90 (Table S5). A few taxa showed only moderate aggregation. For example, *B. crenatus* was consistently the least aggregated where it occurred (e.g., Sanikiluaq: 0.46; Rankin Inlet: 0.55; Cambridge Bay: 0.66), while *E. salvelini*, *Corynosoma* spp., and *D. varicus* exhibited moderate aggregation (D ≈ 0.60–0.79) at some sites (Table S5).

The least abundant parasite taxa were *Podocotyle sinusacca* (1 host, 1 parasite), *Bothriocephalus* spp. (1 host, 2 parasites), *Steringotrema* spp. (2 hosts, 2 parasites) and *Neoechinorhynchus (Neoechinorhynchus) tumidus* (*syn. Neoechinorhynchus tumidus*, 2 hosts, 2 parasites, Table 2). *Podocotyle sinusacca* (found only in Cambridge Bay) and the genera *Bothriocephalus* spp. (found only in Sanikiluaq) and *Steringotrema* spp. (found in Rankin Inlet and Sanikiluaq) to our knowledge, are new records of parasites for Arctic char, at least in Arctic Canada. Other parasite taxa that also appear to be newly described for Arctic char in this region, include *Ascarophis* spp. (4 hosts, 11 parasites, found in Naujaat), *Podocotyle angulata* (4 hosts, 34 parasites, found in Sanirajak and Sanikiluaq) and *Raphidascaris (Raphidascaris) acus* (syn. *Raphidascaris acus*, 9 hosts, 388 parasites, found only in Sanikiluaq, Table 2).

**Fig. 6:**
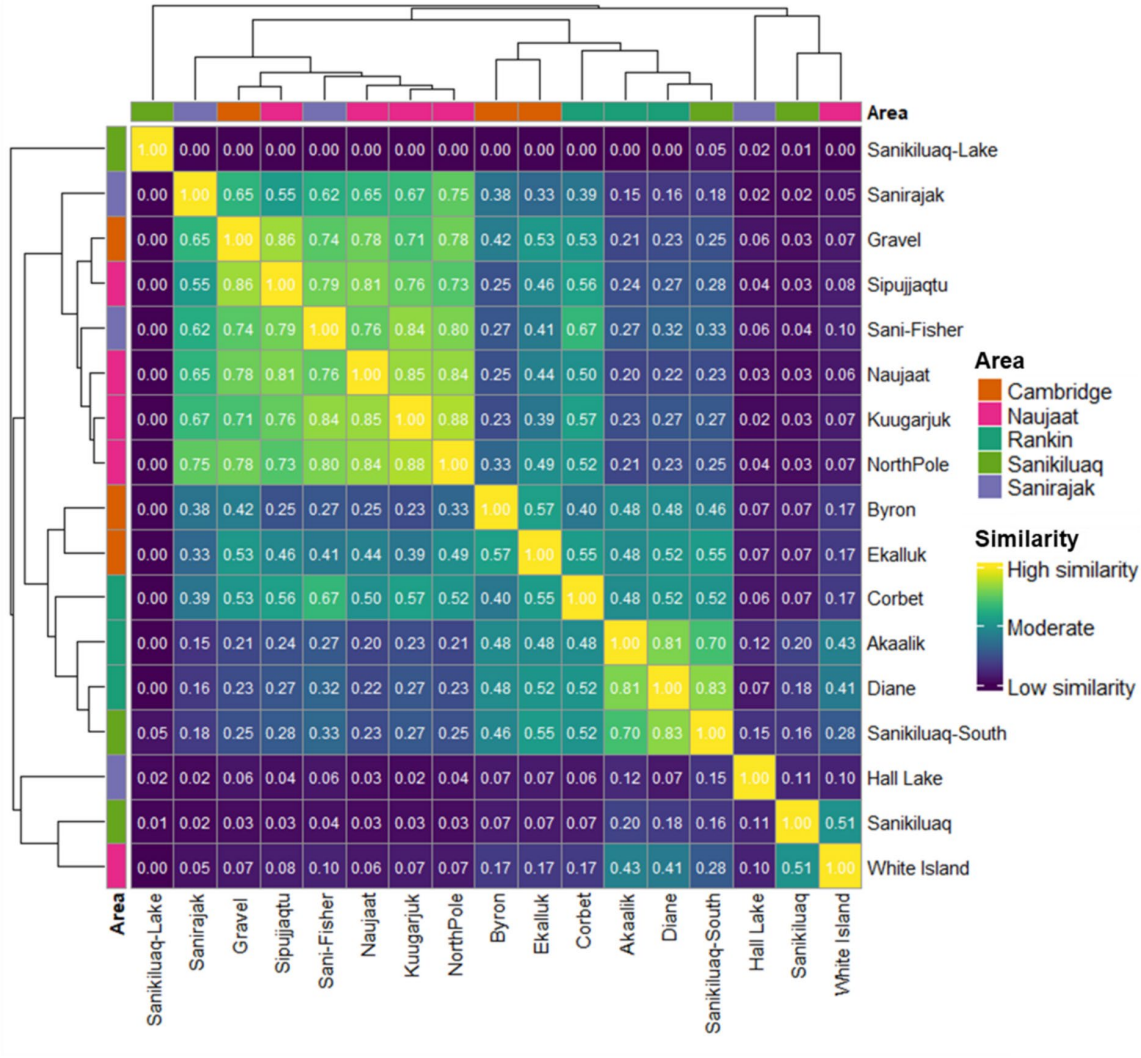
Heatmap of Bray–Curtis similarity in parasite community composition among sampling locations. Parasite abundance data were aggregated by location, and pairwise Bray–Curtis similarity values (1 –dissimilarity) were calculated to assess compositional overlap in parasite assemblages. Rows and columns represent sampling locations, hierarchically clustered using complete linkage based on Bray–Curtis similarity. Cell shading corresponds to pairwise similarity values, and numerical values within cells indicate the corresponding similarity between locations. Sampling areas are indicated by colored annotation bars along the rows and columns

**Fig. 7:**
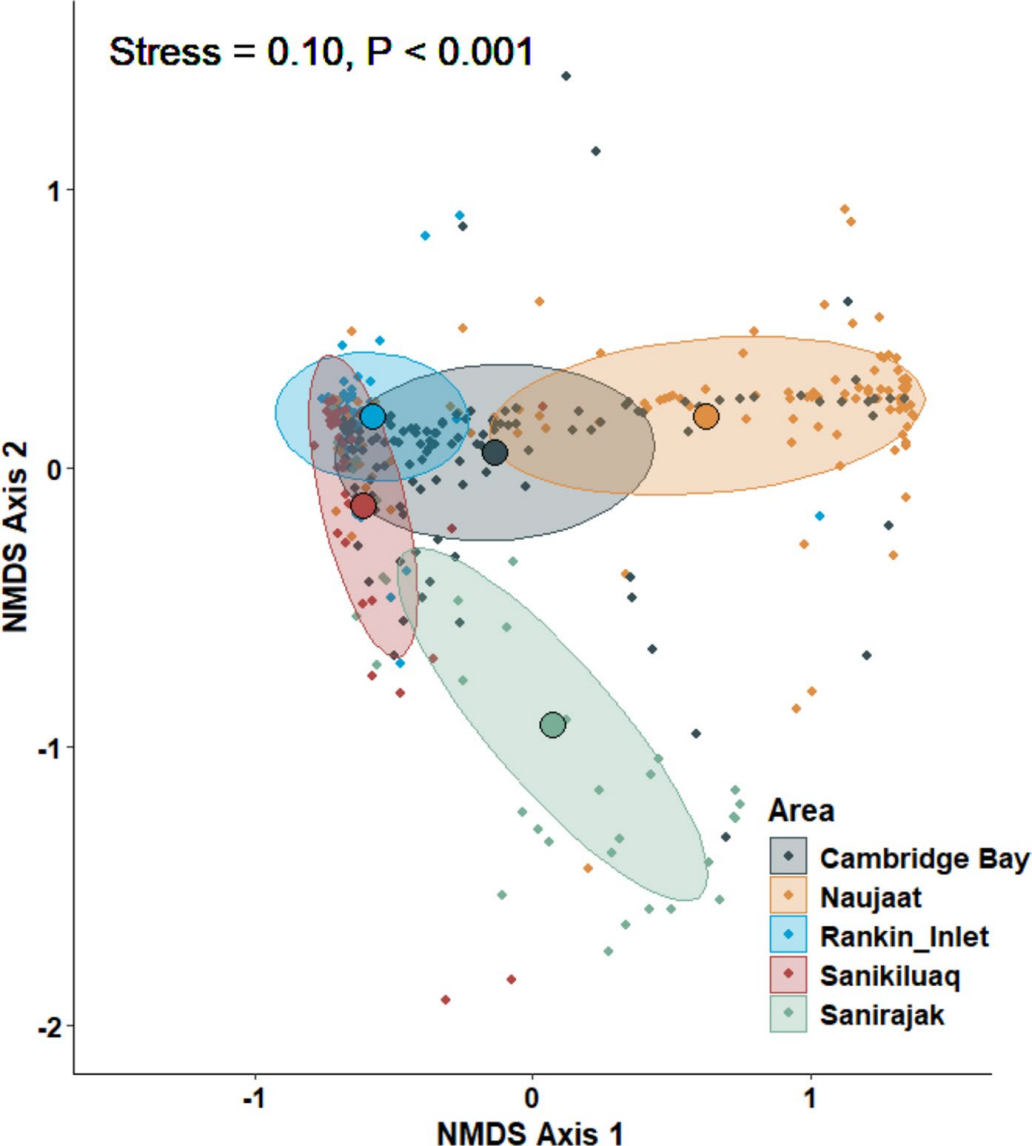
Nonmetric dimensional scaling plots showing differentiation of parasite communities among contemporary Arctic char samples collected from several areas in Nunavut including the Cambridge Bay, Naujaat, Rankin Inlet, Sanirajak and Sanikiluaq areas of the territory. A three-dimensional Bray–Curtis dissimilarity index was used resulting in a stress level of 0.10

Parasite community composition varied markedly among sampling locations (as evidenced by the Bray–Curtis similarity Heatmap), with strong regional clustering evident in several areas (Fig. 6). Notably, Gravel Pit (in Cambridge Bay), Sanirjak, Naujaat sites (Naujaat, Kuugarjuk, North Pole River and Sipujaqtu) exhibited relatively high similarity despite being geographically distant. In contrast, locations separated by major geographic barriers—particularly comparisons between Sanikiluaq and western Kitikmeot sites—showed consistently low similarity. Parasite communities were also compared among sampling areas through NMDS ordinations. NMDS results suggest some heterogeneity in parasite communities based on separation among most sampling areas (Fig. 7). There was, however, significant variability in the parasite communities among sampling areas as indicated by the PERMANOVA (stress = 0.10, p = 0.01) results. Sanirajak and Naujaat sampling areas had the most distinctive parasite communities compared to all other sampling areas (Fig. 7**)**. Rankin Inlet and Sanikiluaq appear to have the most similar parasite communities. Most areas, however, did show some degree of overlap.

### Historical and empirical species overlap

A total of 48 parasite taxa identified to species level were retained from the historical literature, and 23 were retained from the empirical assessment (Figure S4). Of these, 16 species were shared between the historical literature review and the empirical assessment. Shared species included *Alloopistholecithum gibbosum* (syn. *Lecithaster gibbosus*), *Bothrimonus sturionis*, *Brachyphallus crenatus*, *Crepidostomum farionis*, *Cystidicola farionis*, *Derogenes varicus*, *Echinorhynchus gadi*, *Echinorhynchus leidyi*, *Eubothrium salvelini*, *Hemiurus levinseni*, *Hysterothylacium aduncum*, *Prosorhynchus squamatus*, *Pseudocapillaria salvelini* (syn. *Pseudocapillaria (Ichthyocapillaria) salvelini*), *Salmincola carpionis*, *Salmincola edwardsii*, and *Salmonema ephemeridarum*. The historical literature contained 30 species not detected in the empirical assessment, whereas 7 species were observed only in the empirical assessment. Empirical-only species included *Corynosoma wegeneri*, *Echinorhynchus salmonis*, *Ichthyocotylurus erraticus*, *Neoechinorhynchus tumidus*, *Podocotyle angulata*, *Podocotyle sinusacca*, and *Raphidascaris acus*.

Among parasite species shared between the historical literature and the present empirical assessment, prevalence estimates varied widely across both datasets (Table S6). Several taxa remained consistently common in the contemporary survey, including *Brachyphallus crenatus* (83.6%; 50.0–98.4% among areas), *Derogenes varicus* (75.9%; 50.0–95.2%), *Bothrimonus sturionis* (68.0%; 17.7–94.8%), and *Eubothrium salvelini* (54.8%; 15.7–88.2%), with values generally overlapping historical ranges (Table S6). In contrast, pooled contemporary prevalence was comparatively low for *Hemiurus levinseni* (9.0%), *Prosorhynchus squamatus* (6.6%), *Pseudocapillaria (Icthyocapillaria) salvelini* (0.8%), and *Salmonema ephemeridarum* (1.6%) relative to several historical reports (Table S6). Some taxa exhibited higher contemporary prevalence than previously reported, including *Echinorhynchus gadi* (52.9%; historical 0.3–30.9%) and *Hysterothylacium aduncum* (16.1%; historical 1.1–3.0, Table S6). Marked regional heterogeneity was evident for many shared species, particularly *Bothrimonus sturionis*, *Crepidostomum farionis*, *Cystidicola farionis*, and *Echinorhynchus gadi*, whose prevalence varied several-fold among sampling areas.

## Discussion

Arctic regions are undergoing rapid climate-driven transformations (Barber et al. 2008; Lehnherr et al. 2018), profoundly affecting aquatic ecosystems and their taxa (Rühland et al. 2023). Warming has also facilitated the range expansion of sub-Arctic marine species, including parasites and their hosts into the Arctic (Galaktionov 2017). Understanding climate change effects on species and parasite biodiversity requires robust baseline data, but Arctic aquatic ecosystems lack comprehensive information due to difficult weather conditions, high costs to access sites, and other logistical challenges (Bilous et al. 2024). Arctic char, a key subsistence and commercial species, serves as a model for monitoring the impacts of climate change on Arctic ecosystems (Lehnherr et al. 2018).

This review synthesized historical and current knowledge on Arctic char parasites, covering their diversity, abundance, prevalence, and intensity in Canadian populations, and presents new data from populations across Nunavut. The literature review encompassed 30 studies (1956–2024) and identified 67 parasite taxa infecting Arctic char in the North American Arctic, with 48 of them identified to species level. Helminths dominated, with nematodes, cestodes, trematodes, and acanthocephalans being the most commonly encountered taxa. Eighty-two percent of taxa occurred in anadromous char and 54% in freshwater char, including 22 species exclusive to anadromous fish. *Eubothrium salvelini* was the most frequently reported parasite, while *Hemiurus levinseni* showed the highest prevalence, intensity, and abundance; *Pomphorhynchus* spp. and *Phocanema decipiens* were the rarest. These results differ somewhat from our empirical assessment, which identified 35 parasite taxa, including 23 parasites identified to species, six of which have not previously been reported from fish in this region. Our samples included several widespread parasites including *B. crenatus*, *D. varicus, A. gibbosum, P. squamatus* and *C. farionis*, and the cestodes *B. sturionis* and *E. salvelini* as well as less common species including the novel reports mentioned above. The findings from our historical literature review combined with the contemporary samples establish a critical baseline for understanding parasite biodiversity, aiding predictions of climate change impacts on Arctic aquatic taxa and the ecosystems they inhabit in North America.

### Historical and empirical species overlap

Comparison of parasite species shared between the historical literature and the present assessment (**n = 16**) suggests long-term persistence of several core taxa, although direct prevalence comparisons should be interpreted cautiously given differences in geography, season, host populations, and sampling design among studies. Several widespread parasites remained common in the contemporary survey, including *Brachyphallus crenatus* (50.0–98.4% among regions; historical 12.8–99.0%), *Bothrimonus sturionis* (17.7–94.8%; historical 0.2–99.3%), and *Eubothrium salvelini* (15.7–88.2%; historical 3.7–85.0%), indicating continued importance of established transmission pathways. In contrast, pooled prevalence of *Hemiurus levinseni* (9.0%) and *Prosorhynchus squamatus* (6.6%) was lower than several historical reports, while *Echinorhynchus gadi* (52.9%) and *Hysterothylacium aduncum* (16.1%) exceeded previously reported values. Marked variation among contemporary sampling regions for species such as *Cystidicola farionis*, *Crepidostomum farionis*, and *E. gadi* further indicates that local ecological conditions strongly influence infection dynamics. These patterns likely reflect a combination of spatial heterogeneity, shifts in prey and intermediate host communities, ongoing climate-driven ecosystem change, and methodological differences among studies rather than simple directional increases or decreases through time.

### Common species noted

According to our literature review, the two most studied/documented parasites have been *E. salvelini* and *Dibothriocephalus* (syn. *Diphyllobothrium*) spp., both cestodes that use copepods as intermediate hosts, though the latter can also be transferred paratenically from forage fish to larger piscivores. These parasites are found in both anadromous and freshwater Arctic char; *E. salvelini* as adults typically in the foregut/caecae and *Diphyllobothrium* spp. as plerocercoids in the stomach or encysted on the gut lining or on the surface of other organs. Our screening of contemporary fish samples also found high prevalence of *E. salvelini* in all of the studied regions. The research interest in these parasites is likely due to the negative impacts infections can have on salmonid fisheries and aquaculture. These parasites can reduce growth, swimming performance, the ability to adapt to environmental stressors and survival in fish hosts (Boyce and Clarke 1983; Rodger 1991; Poulin et al. 1992; Gallagher et al. 2009). In addition, *Dibothriocephalus* spp. can pose a risk to human health, which may also explain the considerable study interest (Poulin et al. 1992). However, these were not the most abundant parasites identified. On average, the three parasite species with the highest abundance and intensity of infection are the trematodes *D. varicus, H. levinseni* and *B. crenatus,* where the last two are found only in anadromous fish according to the studies identified in the literature review.

Our results are consistent with those of Dick (1984), who found a dominance of digenean trematodes (*Brachyphallus* spp.*, Derogenes* spp., *Lecithaster* [syn. *Alloopistholecithum*] spp. and *Prosorhynchus* spp.) in the parasitic fauna of anadromous Arctic char in North America. According to Dick (1984), the high dominance of these parasites could be explained by the diet, which includes marine amphipods and mollusks, common intermediate hosts for these parasites (Krupenko et al. 2020). Although speculative, the similarities between our contemporary samples and those assessed in 1984 indicate relative stability in the prevalence and abundance of these taxa in this region through time. We suggest that continued monitoring of Arctic char from the North American Arctic for these trematodes could be useful given that some research has suggested that parasite species with complex lifecycles are among those most sensitive to warming climates (Wood et al. 2023). Declines in the prevalence or abundance of these historically common parasites may serve as a future warning signal of change in aquatic Arctic communities more broadly.

Our empirical assessment documented several parasites previously reported from Arctic char across Arctic Canada (this study). Dominant species with > 50% prevalence included *Brachyphallus crenatus*, *Derogenes varicus*, *Bothrimonus sturionis*, *Eubothrium salvelini*, *Echinorhynchus gadi*, and *Alloopistholecithum gibbosum*; all occurred in every region except *E. gadi*, which was absent from Sanikiluaq. *Crepidostomum farionis* and *Prosorhynchus squamatus* were also widespread. All of these parasites infect the stomach and/or intestine of the fish host. These parasites use amphipods (e.g., *Themisto* spp., *Onisimus* spp., *Gammarus* spp.), mysids (*Mysis* spp.), and copepods (*Calanus* spp.) as intermediate (parasite undergoes developmental stages, does not reach reproductive maturity) hosts heavily preyed on by Arctic char (Dempson et al. 2002; Spares et al. 2012; Faulkner et al. 2025), facilitating infection as final (parasite reaches reproductive maturity) or paratenic (a transport host, parasite does not develop further, remains infective until reaching its final host) hosts. Such invertebrates are broadly distributed in the Arctic; *T. libellula*, *O. litoralis*, and *Gammarus* *wilkitzkii* occur in all sampled regions (Dunbar 1957; Vader et al. 2015) and are common in Arctic char diets (L.N. Harris, unpubl.; Faulkner et al. 2025). Widely distributed fishes such as Arctic cod (*Boreogadus saida*) and marine sculpins (*Myoxocephalus* spp.), intermediate or paratenic hosts for *B. crenatus* and *D. varicus*, are likewise frequent Arctic char prey and were recorded in stomachs at all sites (Dempson et al. 2002; Spares et al. 2012; Côté et al. 2021; Faulkner et al. 2025). The broad availability of these prey species likely explains the consistent presence of these common parasites across our study area. Once again, monitoring and comparing the prevalence or abundance of these now common infections in future studies may provide valuable indicators for aquatic community changes more broadly.

### Previously not reported and rare species identified

*Podocotyle angulata* and *P. sinusacca*, that we report here as new records for Arctic char, are common in the intestines of fishes from the coastal North Atlantic and northeast Pacific. These species were differentiated based on differences in sucker ratios, cirrus sac length, testes arrangement, vitelline field distribution, egg size, body proportions, and habitat preference. *Podocotyle* spp. use intertidal Littorinidae gastropods as first intermediate hosts, gammarid amphipods as second intermediate hosts, and various fishes as final hosts. In the Atlantic, *P. angulata* infects brook trout (*S. fontinalis*; Gibson 1996), whereas *P. sinusacca* typically infects Pacific Cottoidei, pleuronectids, and gadids (Morado and McFee 1996; Gibson 1996) and, prior to this study, was not recorded in salmonids. *P. angulata*, previously unknown in the Canadian Arctic, is closely related to *P. atomon* reported in Greenland Arctic cod (*Boreogadus saida*; Køie 2009) and shorthorn sculpin (*Myoxocephalus scorpius*) from Frobisher Bay (Dick et al. 2009). Its broad host range, presence in brook trout, and range into the eastern Canadian Arctic make its occurrence in Arctic char unsurprising and suggest additional hosts may surface. While it is impossible to know whether this new record results from increased sampling or range expansion of the parasite potentially resulting from changing environmental conditions, the latter hypothesis is plausible and argues for continued parasite monitoring efforts in the region. Given *Podocotyle’*s phenotypic variability, however, molecular and morphological analyses are recommended for taxonomy and phylogenetics (Krupenko et al. 2020; Sokolov et al. 2024).

The *Steringotrema* spp. trematode in Arctic char, though poorly preserved, was likely *S. pagelli* or *S. ovacutum*, both occurring along the Labrador coast. *Steringotrema* spp. was identified based on the shape and location of the vitellarium (to Subfamily) and shape of the excretory vesicle (to Genus). These species typically use bivalves and brittle stars as first intermediate hosts and flatfish (Pleuronectidae) or wolffish (Anarhichadidae) as final hosts, suggesting that Arctic char is an incidental host. The nematode *Raphidascaris (Raphidascaris) acus*, common in northern pike (*Esox lucius*), burbot (*Lota lota*), and brook trout, has a Holarctic distribution (Poole and Dick 1984). *R. acus* was identified based on the excretory system/pore shape (Family) and location and the lack of an intestinal caecum (most closely related genus, *Hysterothylacium*, has an intestinal caecum). *R. acus* is also the only known species in the genus in Canada. Larvae infect aquatic insects, gammarids, and forage fish such as yellow perch (*Perca flavescens*). Arctic intermediate hosts are unknown, but its life cycle likely mirrors freshwater systems in southern populations. Although rarely reported in marine fish, it could infect anadromous Arctic char that spend most of the year in freshwater; its prevalence and transmission dynamics in Arctic ecosystems therefore warrant further study.

*Ascarophis* spp., widespread on all Canadian coasts (Arai and Smith 2016), infect gadids and pleuronectids; *A. filiformis* occurs in Arctic cod (*Boreogadus saida*) in Nunavut and Greenland (Køie 2009). Intermediate hosts are typically decapods. The specimens we observed in Arctic char stomachs likely arrived paratenically via infected prey such as Arctic cod, though the persistence of these parasites in Arctic char is uncertain. *Bothriocephalus* spp., primarily freshwater tapeworms of cyprinids (Choudhury and Cole 2012), include *B. cuspidatus* in walleye (*Sander vitreus*) and deepwater sculpin (*Myoxocephalus thompsonii*) in Great Slave Lake and Hudson Bay (Carney et al. 2009). The marine *B. scorpii* infects pleuronectids, gadids, and scorpaenids along North Atlantic and Pacific coasts (Gibson 1996) and has been reported in fourhorn sculpin (*Myoxocephalus quadricornis*) in the western Canadian Arctic (M. Johnson pers. obs., DFO unpubl.). Given these infection patterns, the immature *Bothriocephalus* we recovered from Arctic char could represent *B. scorpii* or a freshwater species. Clarifying its host range and identifying intermediate hosts of all these previously unreported Arctic char parasites in northern waters would help determine whether Arctic char are occasional carriers or a meaningful component of these parasite’s life cycle. Moreover, understanding the environmental and ecological drivers of the occurrence of these parasites in anadromous fishes may provide insight into broader patterns of parasite exchange across freshwater–marine ecosystems.

In addition to the previously unreported and rare genera/species of Arctic char parasites we note above, we identified several more taxa that were not noted at all locations or were rare in general. For example, other rare observations (parasites found in less than 10 hosts) of parasites we could identify to the species level included *Neoechinorhynchus (Neoechinorhynchus) tumidus, Ichthyocotylurus erraticus, Corynosoma wegeneri, Pseudocapillaria (Icthyocapillaria) salvelini* (*syn. Pseudocapillaria salvelini*)*, Hysterothylacium* spp., *Salmincola* spp.*, Crepidostomum* spp.*, Echinorhynchus salmonis, Salmonema ephemeridarum, Philonema* spp., all of which have previously been documented in the geographical range of Arctic char. Of these, only S. *ephemeridarum* has been previously noted in Canadian populations of Arctic char. *I. erraticus* encysts as a metacercaria on the heart of the fish host while *C. wegeneri* is usually found encysted on the viscera or in the mesenteries. Both infect piscivorous birds or marine mammals as adults. *Philonema* spp. can be found free-living in the body cavity of the host. The remainder of the above parasites are typically found in the digestive tract. Increasing sample sizes and the spatial coverage over the known distribution of Arctic char would allow us to further evaluate the distribution and prevalence of these rarer species in Canadian populations of Arctic char and increase our overall understanding of differences in parasite communities among locations.

### Differences in parasite communities among sampling areas

Although the composition of common parasite species was similar across sampling sites in our study, there were notable differences among areas in parasite composition (i.e., types of parasites present), as well as in infection prevalence and intensity (Table 2, Tables S3–S5). For example, excluding the rare or previously unreported species noted above, common species such as *B. crenatus* (prevalence range among areas: 50.0–98.4%), *D. varicus* (50.0–95.2%), *B. sturionis* (17.7–94.8%), *E. salvelini* (15.7–86.8%), *E. gadi* (0–90.3%) and *A. gibbosum* (35.5–68.2%) varied drastically in prevalence among sampling areas (Table S2). Transmission of Arctic char parasites primarily depends on specific intermediate hosts, such as amphipods, copepods, or small fishes, being consumed by Arctic char (Dempson et al. 2002; Côté et al. 2021; Faulkner et al. 2024). The availability and diversity of these hosts can vary geographically due to differences in environmental conditions, such as water temperature, marine ice conditions and salinity, and differences in habitat structure (Węsławski et al. 2011). Thus, areas with a greater diversity of prey and intermediate hosts may harbor a wider array of parasite species, leading to regional differences in parasite communities. The timing and location of sampling may also influence the parasite fauna detected in a sample as the same environmental conditions may vary seasonally and annually and/or along the migration route influencing prey availability and potential recovery from parasites (Shaw and Binning 2016; Faulkner et al. 2025). Additionally, variation in sampling effort among areas can bias parasite assessments, as lower sample sizes may reduce the likelihood of detecting rare taxa, limit the completeness of parasite inventories, and distort quantitative estimates of infection (Vigneault et al. 2025). Together, these factors underscore the importance of considering ecological variability and sampling consistency when interpreting spatial patterns in parasite diversity and prevalence among Arctic char populations.

### Biases and gaps in our understanding

In both the literature review and our empirical data, our results suggest that parasite prevalence can be high in several of the species noted in some regions, with 100% of sampled individuals infected in several cases. These high prevalence values come with a caveat: in several of the past studies, a prevalence of 100% is associated with a small sample size (i.e. 1–10 individuals), indicating a possible overestimation of the actual infection levels. Thus, in some cases, this variable is not necessarily representative of actual host-parasite interactions in a given environment, especially if sampling techniques are biased in favour of certain types of infection over others (Wilson et al. 1993; Thambithurai et al. 2022). Indeed, a recent study on black spot disease caused by trematodes in littoral freshwater fishes demonstrated that low sampling efforts tend to overestimate the prevalence of infection (Vigneault et al. 2025). Although reports of high infection prevalence in a population can be concerning, especially if historic records suggest an increase in prevalence over time, it is important to consider the influence of sampling bias on the results before conclusions regarding the infection levels in a population are drawn.

Dick (1984) first suggested that most studies on Arctic char parasites in North America at that period had not surveyed for protozoa, which could explain the low number of protozoan species identified in Arctic char in the past. Although interest in metazoan parasites, particularly helminths, is still quite strong, our literature review suggests that a broader diversity of parasite taxa have in fact been explored with one taxa of Chromista, two of Fungi and five of Protozoa reported (Fig. 2). The low number of bacteria and viruses identified is likely because these were not targeted by researchers. Current data on bacteria in North American Arctic char come from a 2020 study (Chapman 2020) identifying three species of bacteria. Because sampling and identifying microparasite infections requires different equipment and expertise, including them may not be feasible in studies not explicitly targeting microparasites. This challenge is problematic given the highly pathogenic nature of many microparasitic infections in fishes (Buchmann 2015). A further limitation is that samples were collected across multiple years and sometime across different seasons within a year, and temporal variation in parasite communities was not explicitly accounted for. Interannual and seasonal differences in environmental conditions, host dynamics, and intermediate host availability may influence parasite transmission, meaning that some observed spatial patterns may also reflect temporal variability. Future work with consistent multi-year and multi-seasonal sampling would help disentangle these effects.

### Impacts of a warming Arctic on parasites and human health

Climate change is expected to significantly influence the diversity and prevalence of Arctic char parasites in the Canadian Arctic through a variety of interconnected mechanisms (Hoberg et al. 2013). As Arctic waters warm, the northward expansion of temperate-associated species, including prey, competitors, and intermediate hosts, is already being documented, a phenomenon often referred to as the “borealization” of Arctic marine habitats (e.g., Fossheim et al. 2015; McNicholl et al. 2021; Falardeau et al. 2022). More temperate-associated fish species such as capelin (*Mallotus villosus*) and sand lance (*Ammodytes* spp.), known prey for Arctic char (Dempson et al. 2002, Faulkner et al. 2025), are expanding further north into Arctic regions and could serve as new intermediate hosts for parasite transmission. Competitors and predators of Arctic char, such as Pacific salmon (*Oncorhynchus* spp.), Atlantic salmon (*Salmo salar*) and salmon shark (*Lamna ditropis*) are also expanding their ranges into previously unoccupied or less frequently occupied areas of the Arctic (Biolus and Dunmall 2020; Gallagher and Johnson 2024), leading to further potential changes in parasite-host dynamics. Alongside fish hosts, key intermediate invertebrate hosts, including amphipods and copepods that serve as important prey for Arctic char, are also expanding their range northward (Chan et al. 2019), potentially facilitating the establishment and spread of additional parasitic species in Arctic ecosystems. For example, Littorinid snails, a common intermediate host for several trematode species (e.g., *Podocotyle* spp.) in sub-Arctic intertidal areas have a lower species richness of trematodes in populations at the northern limits of their distribution (Galaktionov 2017). This is likely due to the snail’s low population density in these areas. A warming Arctic could expand the range and densities of Littorinids, enhancing the transmission of *Podocotyle* spp. and potentially other trematode species to new fish hosts.

In addition to shifts in aquatic hosts, migratory birds can act as important vectors for parasite dispersal in Arctic ecosystems, particularly for helminths with complex life cycles involving avian definitive hosts (Galaktionov 1996). Through long-distance movements, birds can transport parasites across regions and introduce them into new freshwater and marine systems used by Arctic char (Galaktionov 1996). Climate-driven changes in migration patterns may further increase host overlap and transmission opportunities. Consequently, bird-mediated dispersal represents an additional pathway by which parasite communities in Arctic char may shift under ongoing environmental change. These changes underscore the likelihood of shifts in parasite communities in Arctic char as the climate warms and species interactions change. The ecological implications of such changes may extend beyond Arctic char to affect entire aquatic ecosystems, as parasites play critical roles in food web dynamics and ecosystem health (Lafferty et al. 2006). With this study providing a baseline of Arctic char parasites across diverse North American regions, continued monitoring will be essential for tracking these shifts and understanding the broader impacts of climate change on Arctic char parasite biodiversity and ecosystem functioning.

Of the 35 parasite taxa identified in Arctic char across Nunavut, only *Dibothriocephalus* spp. and *Anisakis* spp. and *Contracaecum* spp. (in the Anisakinae family) pose potential risks to humans. *Dibothriocephalus* infections are usually mild or asymptomatic, while *Anisakis* can cause more severe gastrointestinal illness (Sakanari and McKerrow 1989; Scholz et al. 2009; Jenkins et al. 2013). Humans serve as definitive hosts for *Dibothriocephalus*, whose life cycle progresses when eggs are consumed by copepods or crustaceans (first intermediate host), then to fishes (second intermediate hosts), and finally to birds or mammals as final hosts. Freshwater species *D. dendriticus*, *D. ditremum*, and *D. latum* have been recorded in resident Arctic char, with *D. dendriticus* and *D. ditremum* confirmed in Lake Hazen, Nunavut (Gallagher et al. 2009). *Anisakis* spp., previously reported in Arctic char from Nunavik and Alaska (Pufall et al. 2012), infect invertebrates as intermediate hosts, then marine fish or cephalopods as paratenic hosts, and reach maturity in marine mammals. Humans are accidental hosts to *A. simplex*, the species most often causing the disease anisakiasis, which occurs in fish, including Arctic char, as larvae (Klimpel et al. 2004). Human cases remain rare, with only nine reported in the North American Arctic between 1982 and 2012 (Pufall et al. 2012). Proper cooking or freezing fish at − 20 °C for seven days or flash-freezing at − 35 °C for 15h prior to consumption prevents infection in humans (Craig 2012; Ljubojevic et al. 2015).

Although human parasitic infections following the consumption of Arctic char are infrequent (Scholz et al. 2009; Bhat and Cleland 2010; Jenkins et al. 2013), climate change may alter the prevalence and distribution of human disease-causing parasites by shifting the ranges of intermediate and definitive hosts (Marcogliese 2008). For example, declines in capelin (*Mallotus villosus*) and Atlantic cod (*Gadus morhua*) have reduced *A. simplex* prevalence in Labrador (Khan and Chandra 2006), whereas northward expansion of Pacific (*Oncorhynchus* spp.) and Atlantic salmon (*Salmo salar*; Dunmall et al. 2013, 2024; Bilous and Dunmall 2020) could introduce previously unreported parasites such as *D. ursi* or increase *A. simplex* in Arctic waters (Curtis 1984; Marcogliese 2001; Karl et al. 2011; Pufall et al. 2012). In addition to direct health related concerns, parasites can affect the real or perceived quality of the fish, and it is common for heavily parasitized fish to be discarded out of caution. Ongoing monitoring is needed to assess climate-driven risks to both Arctic char and human health.

## Conclusion

By conducting a systematic review of North American literature and integrating it with a comprehensive empirical assessment across a vast Canadian Arctic sampling area, we have compiled the most comprehensive understanding of the diversity, prevalence, and distribution of anadromous Arctic char parasites in the region to date. This combined approach offers a nuanced picture of the parasites known to affect this species in this region, helping to fill gaps in our baseline understanding of Arctic char parasite fauna. Our findings serve as a critical baseline for future studies, particularly in light of the rapid environmental changes reshaping Arctic ecosystems. The identification of rare and previously undocumented parasite species raises important questions about their potential to become more prevalent under shifting environmental and ecological conditions, including warming waters, altered host ranges, and changes in prey availability. Furthermore, the knowledge generated here supports broader efforts to predict the impacts of parasitism on Arctic char populations, ensuring the sustainability of this culturally and ecologically significant species. This study not only contributes to the ecological understanding of Arctic char parasites but also underscores the importance of monitoring biodiversity in the face of global climate change. Continued, long-term monitoring of Arctic char parasite communities will be essential for detecting shifts in parasite diversity and prevalence, understanding how environmental change influences aquatic ecosystems, and anticipating potential implications for the people who rely on this species. Monitoring aimed specifically at Arctic char health should also incorporate a broader suite of microparasites and other pathogens not systematically assessed here.

## Supplementary Information

Below is the link to the electronic supplementary material.

Supplementary file1 (DOCX 450 KB)

## Data Availability

Upon acceptance all parasite data will be uploaded to the Open Government Portal.
