# Supplementary material for: Parasites of Arctic char (Salvelinus alpinus) in North America: a systematic literature review and an analysis of contemporary data from anadromous populations from Nunavut
Source: Rev Fish Biol Fish. 2026 Jun 20;36(1):67. doi: 10.1007/s11160-026-10068-x (PMC13283157; doi:10.1007/s11160-026-10068-x)
Supplement: Supplementary file 1 — Supplementary file1 (DOCX 450 KB) [file 11160_2026_10068_MOESM1_ESM.docx]

**Supplementary Material**

**Supplementary Figures**

**
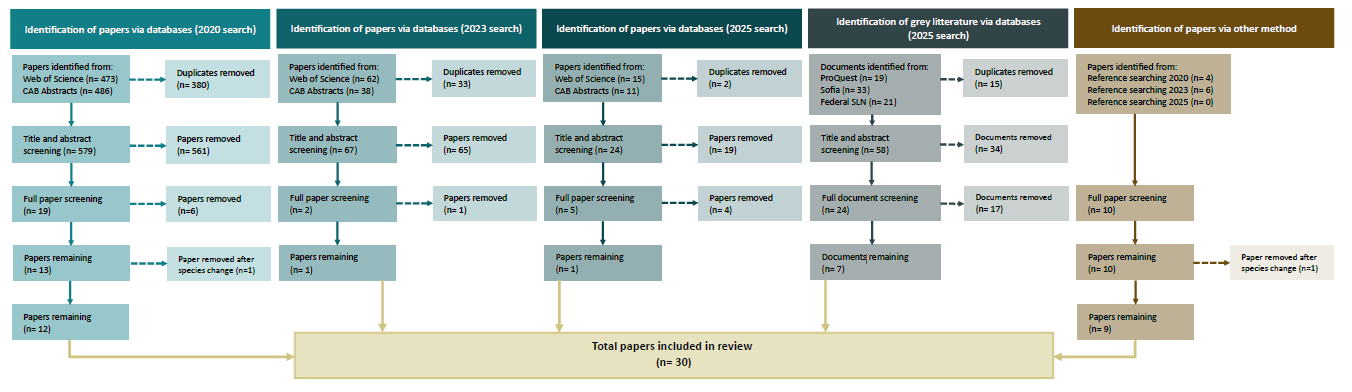
**

**Figure S1.** PRISMA-style workflow summarizing the identification, screening, and inclusion of studies incorporated into the Arctic Char (*Salvelinus alpinus*) parasite literature review. Searches were conducted in 2020, 2023, and 2025 across multiple databases (Web of Science, CAB Abstracts), supplemented with grey-literature searches (ProQuest, Sofia, Federal Science Library Network (SLN)) and reference-list screening. The diagram shows the number of records identified, duplicates removed, titles and abstracts screened, full texts assessed for eligibility, and papers removed after species verification. In total, 30 papers met all criteria and were included in the final review.

**
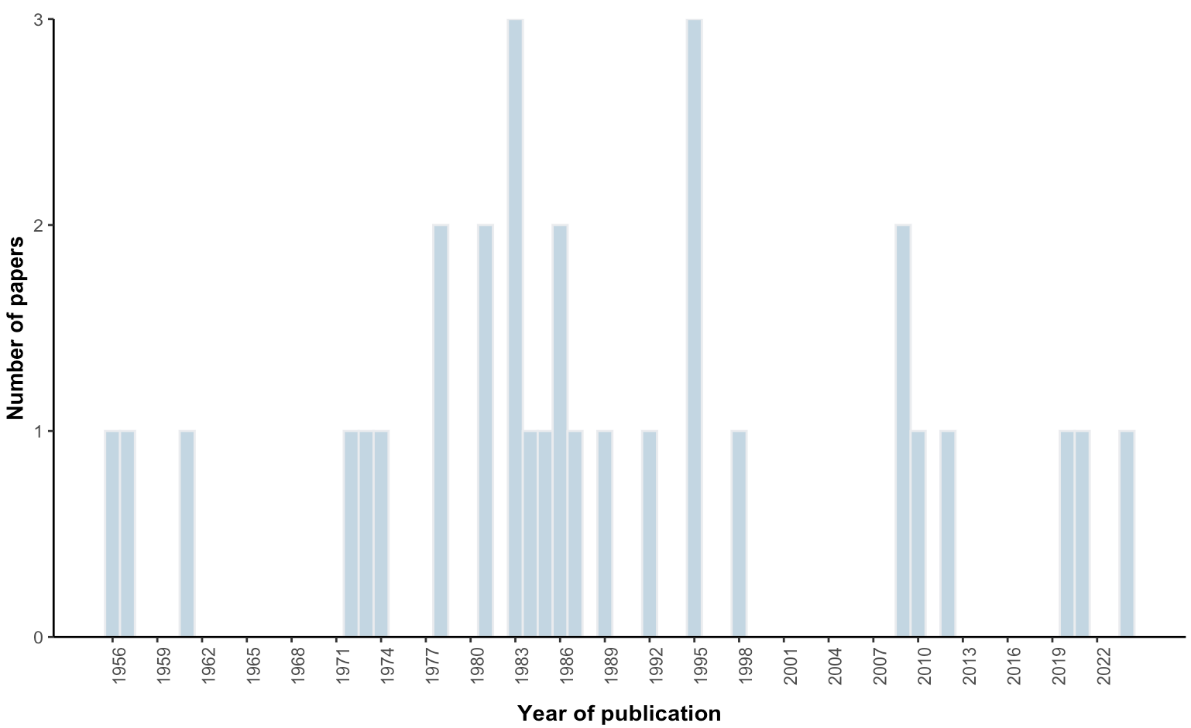
**

**Figure S2**. Histogram of the number of studies containing information on the abundance and/or prevalence of parasites on Arctic char published per year between 1956 and 2024.


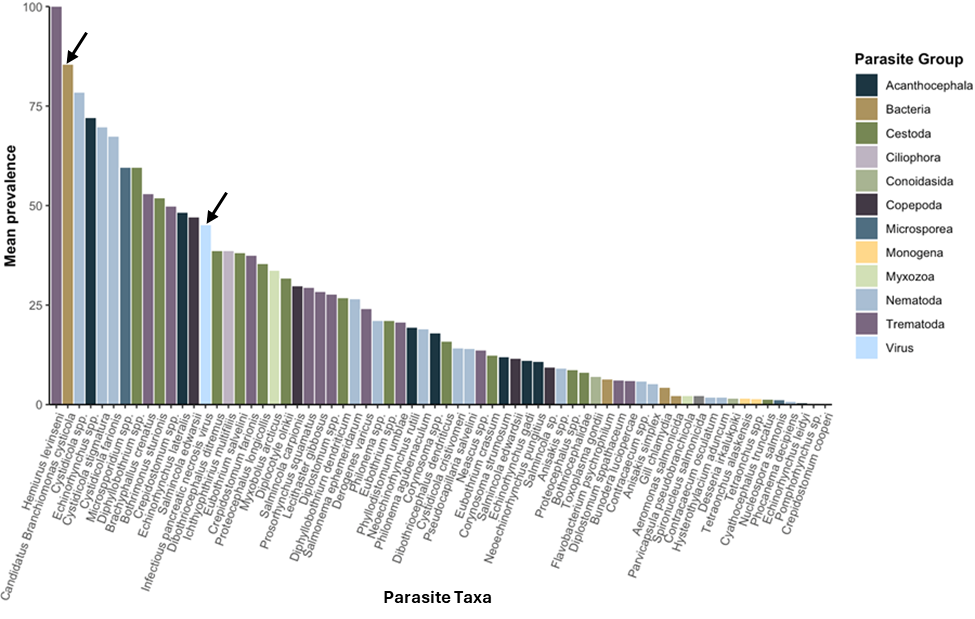


**Figure S3.** Bar plot of the mean prevalence of parasite taxa (also including bacteria and viruses) identified in the systematic literature review. Bacteria and virus taxa are indicated by arrows to distinguish them from parasite taxa.

**
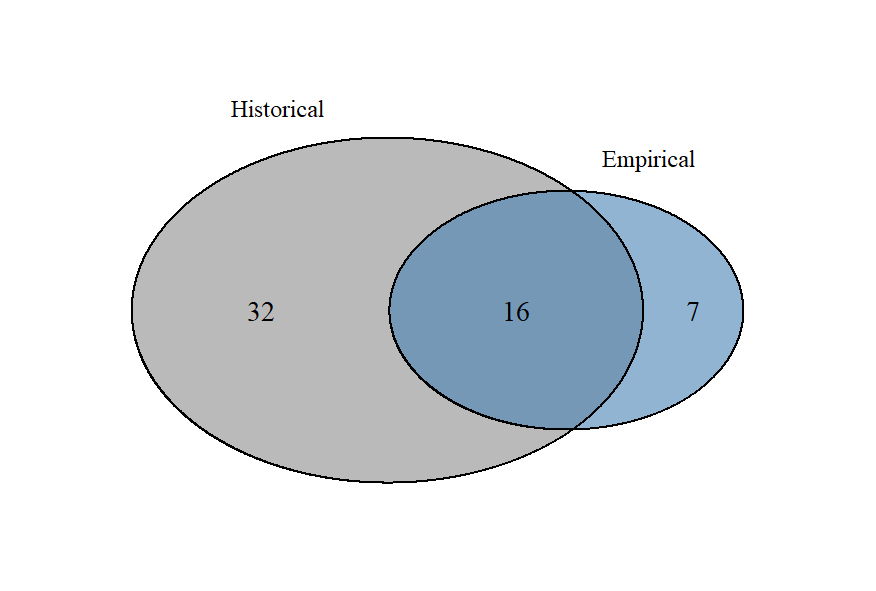
**

**Figure S4.** Comparison of parasite taxa reported from Arctic char in the historical literature and those detected in the present empirical assessment, restricted to records identified to species level to minimize ambiguity associated with genus-level or higher-rank identifications. Bars represent taxa unique to the historical literature, taxa shared between datasets, and taxa unique to the empirical assessment. The Venn diagram illustrates exact overlap in species-level identifications following taxonomic standardization. **Historical only:** *Anisakis simplex*, *Branchiomonas cysticola*, *Bunodera luciopercae*, *Contracaecum osculatum*, *Crepidostomum cooperi*, *Corynosoma strumosum*, *Cyathocephalus truncatus*, *Cystidicola cristivomeri*, *Cystidicola stigmatura*, *Desseria irkalukpiki*, *Dibothriocephalus dendriticus*, *Dibothriocephalus ditremus*, *Diplocotyle olrikii*, *Diplostomum spathaceum*, *Echinorhynchus lateralis*, *Eubothrium crassum*, *Flavobacterium psychrophilum*, *Ichthyophthirius multifiliis*, *Myxobolus arcticus*, *Neoechinorhynchus pungitius*, *Neoechinorhynchus rutili*, *Nucleospora salmonis*, *Parvicapsula pseudobranchicola*, *Philonema agubernaculum*, *Phocanema decipiens*, *Phyllodistomum umblae*, *Proteocephalus longicollis*, *Spironucleus salmonicida*, *Tetraonchus alaskensis*, *Toxoplasma gondii*. **Empirical only:** *Corynosoma wegeneri*, *Echinorhynchus salmonis*, *Ichthyocotylurus erraticus*, *(Neoechinorhynchus) tumidus (syn. Neoechinorhynchus tumidus)*, *Podocotyle angulata*, *Podocotyle sinusacca*, *Raphidascaris (Raphidascaris) acus (syn. Raphidascaris acus)*. **Shared:** *Alloopistholecithum gibbosum (syn. Lecithaster gibbosus)*, *Bothrimonus sturionis*, *Brachyphallus crenatus*, *Crepidostomum farionis*, *Cystidicola farionis*, *Derogenes varicus*, *Echinorhynchus gadi*, *Echinorhynchus leidyi*, *Eubothrium salvelini*, *Hemiurus levinseni*, *Hysterothylacium aduncum*, *Prosorhynchus squamatus*, *Pseudocapillaria salvelini*, *Salmincola carpionis*, *Salmincola edwardsii*, *Salmonema ephemeridarum*.

**Table S1.** Papers identified in the systematic literature review using keyword searches in Web of Science (WOS) and Cab Abstract (CAB) as well as targeted searches of reference lists and grey literature conducted in 2020, 2023 and 2025 using Federal Science Library Networks (SLN) and Sofia. Full references are included at the end of the supplementary material.

|  | **Authors and year** | **Source** | **Search method and year** |
| --- | --- | --- | --- |
| 1 | Andrews and Lear (1956) | Journal of the Fisheries Research Board of Canada | Targeted search 2020 |
| 2 | Bérubé and Curtis (1986) | Canadian Journal of Fisheries and Aquatic Sciences | Targeted search 2023 |
| 3 | Beverley-Burton (1978) | Canadian Journal of Zoology | WOS 2020 |
| 4 | Black and Lankester (1981) | Canadian Journal of Zoology | WOS 2020 |
| 5 | Black (1983a) | Canadian Journal of Fisheries and Aquatic Sciences | CAB 2020 |
| 6 | Black (1983b) | Canadian Journal of Fisheries and Aquatic Sciences | Targeted search 2023 |
| 7 | Black (1985) | Canadian Journal of Zoology | WOS 2020 |
| 8 | Bouillon and Curtis (1987) | Journal of Wildlife Diseases | WOS 2020 |
| 9 | Bouillon and Dempson (1989) | Canadian Journal of Zoology | Targeted search 2023 |
| 10 | Chapman (2020) | Carleton University | Sofia 2025 |
| 11 | Curtis (1983) | International Society of Arctic Char Fanatics | Federal SLN 2025 |
| 12 | Curtis and *al.* (1995) | Canadian Journal of Fisheries and Aquatic Sciences | WOS 2020 |
| 13 | Curtis and Jensen (1992) | Research on Arctic Biology : Igloolik, Northwest Territories, Canada, June 8th - July 8th 1992 | Federal SLN 2025 |
| 14 | Desdevises and *al*. (1998) | Journal of the Helminthological Society of Washington | WOS 2020 |
| 15 | Dick (1984) | Parasites and Arctic charr management - an academic curiosity or practical reality? | Federal SLN 2025 |
| 16 | Dick and Belosevic (1981) | Journal of Fish Biology | Targeted search 2020 |
| 17 | Due and Curtis (1995) | Journal of Fish Biology | WOS 2020 |
| 18 | Eddy and Lankester (1978) | Journal of the Fisheries Research Board of Canada | WOS 2020 |
| 19 | Gallagher and *al*. (2009) | Government Document- Department of Fisheries and Oceans Canada | Targeted search 2020 |
| 20 | Gallagher and Dick (2010) | Ecology of Freshwater Fish | WOS 2020 |
| 21 | Hanek and Molnar (1974) | Journal of the Fisheries Research Board of Canada | Targeted search 2023 |
| 22 | Hicks and Threlfall (1973) | Journal of Fish Biology | Targeted search 2020 |
| 23 | Isinguzo (2009) | University of Manitoba | ProQuest 2025 |
| 24 | Jalenques and *al*. (2021) | Diseases of Aquatic Organisms | WOS 2023 |
| 25 | Jamieson (1972) | University of Toronto | Federal SLN 2025 |
| 26 | Kolasa and Curtis (1995) | Nordic Journal of Freshwater Research | CAB 2020 |
| 27 | Laird (1961) | Canadian Journal of Zoology | Targeted search 2023 |
| 28 | Merks et *al*. (2024) | Zoonoses and Public Health | WOS 2025 |
| 29 | Pufall et *al.* (2012) | Foodborne Pathogens and Disease | WOS 2020 |
| 30 | Thomson (1957) | McGill University | Sofia 2025 |

**Table S2.** Summary of Arctic Char (*Salvelinus alpinus*) parasite taxa reported in the published literature (1956–2024), including mean prevalence, mean intensity, and mean abundance extracted from each reference. Life-history form assessed in each study (ANAD = anadromous; FWR = freshwater resident; ND = not defined) is indicated, along with whether each parasite taxon was also detected in our empirical assessment (“Yes,” “No,” or “Maybe,” where species-level confirmation was uncertain). Values marked with “‡” represent abundance estimates calculated from reported prevalence and intensity. ND indicates data not reported in the original study. Bold taxon names listed below historical species names represent the currently accepted nomenclature.

| **Species/Taxa** | **Mean Prevalence  (%)** | | **Mean Intensity** | **Mean Abundance** | **References** | **Life History  Form  Assessed** | **Identified  in This Study** |
| --- | --- | --- | --- | --- | --- | --- | --- |
| **Acanthocephala** | | | | | | | |
| *Corynosoma* spp*.* | 7.37 | 1.14±0.37 | | 0.08 | Dick and Belosevic (1981) | ANAD | Yes |
|  | 28.38 | ND | | ND | Isinguzo (2009) | ANAD |  |
| *Corynosoma strumosum* | 4.27 | ND | | ND | Jamieson (1972) | ANAD | No |
|  | 19.40 | 1.55±1.20 | | 0.26‡ | Desdevises et al. (1998) | ANAD |  |
| *Echinorhynchus* spp*.* | 72.04 | 59.5±ND | | 43.57 | Dick (1984)^†^ | ND | Yes |
| *Echinorhynchus gadi* | 0.26 | 5.05±6.55 | | 1.86‡ | Desdevises et al. (1998) | ANAD | Yes |
|  | 30.87 | 10.27±15.59 | | 4.45 | Dick and Belosevic (1981) | FWR, ANAD |  |
|  | 7.90 | 1.56±ND | | 0.79 | Due and Curtis (1995) | FWR, ANAD |  |
|  | 11.11 | 2.00±ND | | 0,22‡ | Hanek and Molnar (1974) | ND |  |
|  | 4.95 | ND | | ND | Isinguzo (2009) | ANAD |  |
| *Echinorhynchus lateralis* | 19.35 | 17.65±ND | | 3.73‡ | Bouillon and Dempson (1989)^†^ | FWR, ANAD | No |
|  | 81.00 | ND | | ND | Curtis et al. (1995) | FWR |  |
|  | 26.00 | 1.00±ND | | 0.26‡ | Hicks and Threlfall (1973)^†^ | ND |  |
|  | 66.54 | 18.02±ND | | 12.43 | Kolasa and Curtis (1995) | FWR |  |
| *Echinorhynchus leidyi* | 0.31 | ND | | ND | Jamieson (1972) | ANAD | Yes |
| *Neoechinorhynchus pungitius* | 10.70 | 5.48±0.60 | | 0.56‡ | Gallagher and Dick (2010) | FWR | No |
| *Neoechinorhynchus rutili* | 19.35 | 3.29±3.70 | | 0.56 | Dick and Belosevic (1981) | FWR, ANAD | No |
| *Pomphorhynchus* spp*.* | 0.19 | ND | | ND | Jamieson (1972) | ANAD | No |
| **Cestoda** | | | | | | | |
| *Bothrimonus sturionis* | 69.60 | 144.50±ND | | 100.57‡ | Bouillon and Dempson (1989) | ANAD | Yes |
|  | 49.55 | 10.60±19.55 | | 6.16‡ | Desdevises et al. (1998) | ANAD |  |
|  | 99.30 | 238.55±395.18 | | 241.59 | Dick and Belosevic (1981) | ANAD |  |
|  | 0.20 | 0.29±ND | | 0.00 | Due and Curtis (1995) | FWR, ANAD |  |
|  | 40.00 | 12.00±ND | | 4.80‡ | Hicks and Threlfall (1973) | ND |  |
|  | 51.96 | ND | | ND | Isinguzo (2009) | ANAD |  |
| Bothriocephalidae | 8.02 | ND | | ND | Thomson (1957) | FWR, ANAD | Yes |
| *Cyathocephalus truncatus* | 1.25 | 0.50±0.00 | | 0.06‡ | Gallagher and Dick (2010) | FWR | No |
| *Diphyllobothrium dendriticum* (***Dibothriocephalus dendriticus****)* | 26.67 | ND | | ND | Jamieson (1972) | ANAD | No |
| *Diphyllobothrium spp.*  (***Dibothriocephalus* spp.)** | 43.70 | ND | | ND | Beverly-Burton (1978) | FWR | Yes |
|  | 85.00 | ND | | ND | Curtis (1983) | ND |  |
|  | 48.39 | 20.40±ND | | 10.24 | Dick (1984) | ND |  |
|  | 81.92 | 123.31±129.90 | | 208.29 | Dick and Belosevic (1981) | FWR, ANAD |  |
|  | 56.60 | 230.09±226.62 | | 211.99‡ | Gallagher and Dick (2010) | FWR |  |
|  | 63.62 | 155.48±224.12 | | 141.05 | Gallagher et al. (2009) | FWR |  |
|  | 100.00 | 19.00±ND | | 19.00 | Hanek and Molnar (1974) | ND |  |
|  | 18.81 | ND | | ND | Isinguzo (2009) | ANAD |  |
|  | 72.34 | ND | | ND | Jamieson (1972) | ANAD |  |
|  | 24.30 | ND | | ND | Thomson (1957) | FWR, ANAD |  |
| *Dibothriocephalus dendriticus* | 6.65 | 36.10±ND | | 3.52‡ | Bouillon and Dempson (1989)^†^ | FWR, ANAD | Maybe |
|  | 13.15 | ND | | ND | Curtis et al. (1995)† | FWR |  |
|  | 13.26 | 2.74±ND | | 0.43 | Due and Curtis (1995)^†^ | FWR, ANAD |  |
|  | 55.48 | 100.23±85.20 | | 91.46‡ | Gallagher and Dick (2010)^†^ | FWR |  |
|  | 3.52 | 64.75±80.52 | | 7.53 | Gallagher et al. (2009)† | FWR |  |
|  | 2.35 | 1.5±ND | | 0.04 | Kolasa and Curtis (1995)^†^ | FWR |  |
| *Dibothriocephalus ditremus* | 49.85 | ND | | 48.86 | Bérubé and Curtis (1986)^†^ | ND | Maybe |
|  | 13.15 | 158.60±ND | | 16.34‡ | Bouillon and Dempson (1989)^†^ | FWR, ANAD |  |
|  | 74.50 | ND | | ND | Curtis et al. (1995)† | FWR |  |
|  | 43.16 | 19.44±ND | | 11.91 | Due and Curtis (1995)^†^ | FWR, ANAD |  |
|  | 53.31 | 147.64±175.53 | | 138.48‡ | Gallagher and Dick (2010)^†^ | FWR |  |
|  | 21.48 | 167.42±213.00 | | 44.97 | Gallagher et al. (2009)† | FWR |  |
|  | 14.52 | 2.50±ND | | 0.35 | Kolasa and Curtis (1995)^†^ | FWR |  |
| *Diplocotyle olrikii* | 31.66 | ND | | ND | Jamieson (1972) | ANAD | No |
| *Eubothrium* spp*.* | 8.50 | ND | | ND | Beverly-Burton (1978) | FWR | Yes |
|  | 28.385 | 16.12±ND | | 3.57 | Dick (1984) | ND |  |
|  | 26.00 | 116.00±ND | | 30.16‡ | Hicks and Threlfall (1973) | ND |  |
| *Eubothrium crassum* | 13.65 | 7.00±ND | | 0.69‡ | Bouillon and Dempson (1989) | FWR, ANAD | No |
|  | 11.00 | 10.00±ND | | 1.10‡ | Hicks and Threlfall (1973) | ND |  |
| *Eubothrium salvelini* | 85.00 | ND | | ND | Andrews and Lear (1956)^†^ | ANAD | Yes |
|  | 4.20 | ND | | ND | Beverly-Burton (1978) | FWR |  |
|  | 3.70 | 2.00±ND | | 0.10‡ | Bouillon and Dempson (1989) | FWR, ANAD |  |
|  | 45.90 | ND | | ND | Curtis et al. (1995) | FWR |  |
|  | 48.98 | 1.38±ND | | 0,67‡ | Curtis and Jensen (1992) | FWR, ANAD |  |
|  | 42.85 | 2.50±1.55 | | 1.02‡ | Desdevises et al. (1998) | ANAD |  |
|  | 84.23 | 57.05±55.21 | | 69.45 | Dick and Belosevic (1981) | FWR, ANAD |  |
|  | 62.04 | 21.31±ND | | 16.19 | Due and Curtis (1995) | FWR, ANAD |  |
|  | 30.67 | 28.64±19.73 | | 27.15‡ | Gallagher and Dick (2010) | FWR |  |
|  | 19.90 | 25.35±6.58 | | 5.90 | Gallagher et al. (2009) | FWR |  |
|  | 9.90 | 3.00±ND | | 0.27‡ | Hicks and Threlfall (1973) | ND |  |
|  | 21.72 | ND | | ND | Isinguzo (2009) | ANAD |  |
|  | 11.27 | ND | | ND | Jamieson (1972) | ANAD |  |
|  | 63.05 | 8.60±ND | | 5.26 | Kolasa and Curtis (1995) | FWR |  |
| *Proteocephalus* spp*.* | 13.94 | 2.89±ND | | 0.47 | Dick (1984) | ND | Yes |
|  | 16.00 | 5.00±ND | | 1.32‡ | Hicks and Threlfall (1973) | ND |  |
|  | 2.63 | ND | | ND | Isinguzo (2009) | ANAD |  |
|  | 2.02 | 2.59±ND | | 0.02 | Kolasa and Curtis (1995) | FWR |  |
| *Proteocephalus longicollis* | 16.90 | ND | | ND | Beverly-Burton (1978) | FWR | Yes |
|  | 2.50 | 6.95±ND | | 0.32‡ | Bouillon and Dempson (1989)^†, §^ | FWR, ANAD |  |
|  | 87.24 | 209.46±264.14 | | 227.40 | Dick and Belosevic (1981) | FWR |  |
|  | 22.91 | 36.21±ND | | 26.46 | Due and Curtis (1995) | FWR, ANAD |  |
|  | 45.06 | 31.08±50.69 | | 18.91‡ | Gallagher and Dick (2010) | FWR |  |
|  | 88.89 | 370.00±ND | | 328.89‡ | Hanek and Molnar (1974)^†^ | ND |  |
|  | 14.00 | 9.00±ND | | 1.26‡ | Hicks and Threlfall (1973)^†^ | ND |  |
|  | 5.19 | ND | | ND | Jamieson (1972) | ANAD |  |
| **Ciliophora** | | | | | | | |
| *Ichthyophthirius multifiliis* | 38.54 | ND | | ND | Chapman (2020) | ANAD | No |
| **Conoidasida** | | | | | | | |
| *Desseria irkalukpiki* | 1.55 | ND | | ND | Laird (1961)^†^ | ANAD | No |
| *Toxoplasma gondii* | 6.95 | ND | | ND | Merks et al. (2024)* | ANAD | No |
| **Copepoda** | | | | | | | |
| *Salmincola carpionis* | 20.70 | 1.60±ND | | 0.33‡ | Bouillon and Dempson (1989) | ANAD | Yes |
|  | 61.60 | 6.25±7.95 | | 4.25‡ | Desdevises et al. (1998) | ANAD |  |
|  | 6.81 | 1.13±ND | | 0.20 | Due and Curtis (1995) | FWR, ANAD |  |
| *Salmincola edwardsii* | 2.80 | ND | | ND | Beverly-Burton (1978) | FWR | Yes |
|  | 13.10 | 1.80±ND | | 0.33‡ | Bouillon and Dempson (1989) | FWR, ANAD |  |
|  | 39.33 | 3.13±3.13 | | 1.17 | Dick and Belosevic (1981) | FWR, ANAD |  |
|  | 5.64 | 1.46±ND | | 0.21 | Due and Curtis (1995) | FWR, ANAD |  |
|  | 6.67 | 1.85±1.63 | | 0.45‡ | Gallagher and Dick (2010) | FWR |  |
|  | 11.88 | 1.90±1.80 | | 0.75 | Gallagher et al. (2009) | FWR |  |
|  | 1.00 | 5.00±ND | | 0.05‡ | Hicks and Threlfall (1973) | ND |  |
|  | 46.97 | ND | | ND | Jamieson (1972) | ANAD |  |
| *Salmincola* spp*.* | 9.27 | ND | | ND | Thomson (1957) | FWR, ANAD | Yes |
| **Flagellata** | | | | | | | |
| *Spironucleus salmonicida* | 2.08 | ND | | ND | Chapman (2020) | ANAD | No |
| **Microsporea** | | | | | | | |
| *Microsporidium* spp*.* | 59.50 | ND | | ND | Jalenques et al. (2021) | ANAD | No |
| *Nucleospora salmonis* | 1.04 | ND | | ND | Chapman (2020) | ANAD | No |
| **Monogena** | | | | | | | |
| *Tetraonchus* spp*.* | 1.30 | 1.00±ND | | 0.01‡ | Bouillon and Dempson (1989) | FWR | No |
| *Tetraonchus alaskensis* | 1.40 | ND | | ND | Beverly-Burton (1978) | FWR | No |
|  | 2.70 | 1.00±0.00 | | 0.03‡ | Desdevises et al. (1998) | ANAD |  |
|  | 0.20 | 0.14±ND | | 0.21 | Due and Curtis (1995) | FWR, ANAD |  |
| **Myxozoa** | | | | | | | |
| *Myxobolus arcticus* | 33.60 | ND | | ND | Desdevises et al. (1998) | ANAD | No |
| *Parvicapsula pseudobranchicola* | 2.08 | ND | | ND | Chapman (2020) | ANAD | No |
| **Nematoda** | | | | | | | |
| *Anisakis* spp*.* | 9.00 | 1.00±ND | | 0.09‡ | Hicks and Threlfall (1973) | ND | Yes |
| *Anisakis simplex* | 5.19 | 0.61±ND | | 0.13 | Due and Curtis (1995) | FWR, ANAD | Maybe |
|  | ND | ND | | ND | Pufall et al. (2012) | ND |  |
| *Contracaecum* spp*.* | 13.27 | 9.08±4.15 | | 0.54 | Dick and Belosevic (1981) | ANAD | No |
|  | 1.09 | 0.20±ND | | 0.01 | Due and Curtis (1995) | FWR, ANAD |  |
|  | 3.00 | 1.00±ND | | 0.03‡ | Hicks and Threlfall (1973) | ND |  |
| *Contracaecum osculatum* | 1.80 | 0.31±ND | | 0.01 | Due and Curtis (1995) | FWR, ANAD | No |
| *Cystidicola* spp*.* | 78.36 | 129.65±ND | | 118.43 | Dick (1984) | ND | Yes |
| *Cystidicola cristivomeri* | 14.08 | ND | | ND | Jamieson (1972) | ANAD | No |
| *Cystidicola farionis* | 50.96 | ND | | ND | Black (1983b) | ND | Yes |
| *Cystidicola stigmatura* | 31.00 | ND | | ND | Beverly-Burton (1978) | FWR | No |
|  | 80.74 | ND | | ND | Black (1983a) | ND |  |
|  | 99.88 | ND | | ND | Black (1985) | FWR |  |
|  | 92.35 | ND | | ND | Black and Lankester (1981)^†^ | FWR |  |
|  | 36.89 | 181.22±64.67 | | 110.51‡ | Eddy and Lankester (1978)^†^ | FWR, ANAD |  |
| *Hysterothylacium aduncum* | 1.10 | 1.00±ND | | 0.01‡ | Bouillon and Dempson (1989) | ANAD | Yes |
|  | 1.17 | 0.23±ND | | 0.01 | Due and Curtis (1995) | FWR, ANAD |  |
|  | 3.00 | 5.00±ND | | 0.15‡ | Hicks and Threlfall (1973)^†^ | ND |  |
| *Philonema* spp*.* | 38.50 | ND | | ND | Andrews and Lear (1956) | ANAD | Yes |
|  | 3.60 | ND | | ND | Thomson (1957) | FWR, ANAD |  |
| *Philonema agubernaculum* | 17.25 | 4.65±ND | | 0.79‡ | Bouillon and Dempson (1989) | FWR, ANAD | Maybe |
|  | 38.80 | 2.20±1.45 | | 0.99‡ | Desdevises et al. (1998) | ANAD |  |
|  | 19.77 | 1.92±2.20 | | 0.22 | Dick and Belosevic (1981) | FWR, ANAD |  |
|  | 25.26 | 2.53±ND | | 0.87 | Due and Curtis (1995) | FWR, ANAD |  |
|  | 4.90 | 1.40±0.00 | | 0.15‡ | Gallagher and Dick (2010) | FWR |  |
|  | 11.65 | 1.47±1.10 | | 0.23 | Gallagher et al. (2009) | FWR |  |
|  | 20.00 | 2.00±ND | | 0.40‡ | Hicks and Threlfall (1973) | ND |  |
|  | 5.06 | ND | | ND | Jamieson (1972) | ANAD |  |
|  | 27.56 | 1.29±ND | | 0.29 | Kolasa and Curtis (1995) | FWR |  |
| *Phocanema decipiens* | 0.76 | 0.14±ND | | 0.1 | Due and Curtis (1995) | FWR, ANAD | No |
|  | ND | ND | | ND | Pufall et al. (2012) | ND |  |
| *Pseudocapillaria salvelini*  (***Pseudocapillaria (Icthyocapillaria) salvelini***) | 2.90 | 9.00±ND | | 0.25‡ | Bouillon and Dempson (1989)^†^ | FWR, ANAD | Yes |
|  | 9.11 | 3.86±ND | | 1.13 | Due and Curtis (1995) | FWR, ANAD |  |
|  | 30.00 | 10.00±ND | | 0.30‡ | Hicks and Threlfall (1973)^†^ | ND |  |
| *Salmonema ephemeridarum* | 10.50 | 3.15±ND | | 0.43‡ | Bouillon and Dempson (1989)^†^ | FWR, ANAD | Yes |
|  | 58.90 | 53.20±4.23 | | 5.22 | Dick and Belosevic (1981)^†^ | ANAD |  |
|  | 33.33 | 8.00±ND | | 2.67‡ | Hanek and Molnar (1974)^†^ | ND |  |
|  | 3.00 | 1.00±ND | | 0.03‡ | Hicks and Threlfall (1973)^†^ | ND |  |
| **Trematoda** | | | | | | | |
| *Brachyphallus crenatus* | 68.50 | 77.20±ND | | 52.88‡ | Bouillon and Dempson (1989) | ANAD | Yes |
|  | 99.03 | 578.57±734.71 | | 1111.83 | Dick and Belosevic (1981) | ANAD |  |
|  | 31.00 | 12.00±ND | | 3.72‡ | Hicks and Threlfall (1973) | ND |  |
|  | 12.84 | ND | | ND | Isinguzo (2009) | ANAD |  |
| *Bunodera luciopercae* | 8.70 | 17.60±ND | | 1.27‡ | Bouillon and Dempson (1989) | FWR, ANAD | No |
|  | 3.00 | 1.00±ND | | 0.03‡ | Hicks and Threlfall (1973) | ND |  |
| *Crepidostomum* spp*.* | 49.73 | 19.87 | | 10.45 | Dick (1984) | ND | Yes |
| *Crepidostomum cooperi* | ND | ND | | ND | Hanek and Molnar (1974) | ND | Maybe |
| *Crepidostomum farionis* | 34.30 | 50.05±ND | | 28.65‡ | Bouillon and Dempson (1989) | FWR, ANAD | Yes |
|  | 69.85 | ND | | ND | Curtis et al. (1995) | FWR |  |
|  | 35.75 | 47.17±97.10 | | 25.87 | Dick and Belosevic (1981) | FWR, ANAD |  |
|  | 31.86 | 6.27±ND | | 2.63 | Due and Curtis (1995) | FWR, ANAD |  |
|  | 47.13 | 17.02±27.29 | | 11.39‡ | Gallagher and Dick (2010) | FWR |  |
|  | 14.00 | 1.00±ND | | 0.14‡ | Hicks and Threlfall (1973) | ND |  |
|  | 28.50 | 5.39±ND | | 2.00 | Kolasa and Curtis (1995) | FWR |  |
| *Derogenes varicus* | 17.40 | 2.6±ND | | 0.45‡ | Bouillon and Dempson (1989) | ANAD | Yes |
|  | 100.00 | 5704.75±4278.45 | | 5704.75‡ | Desdevises et al. (1998) | ANAD |  |
|  | 4.21 | 8.75±14.17 | | 0.37 | Dick and Belosevic (1981) | ANAD |  |
|  | 3.37 | 0.70±ND | | 0.09 | Due and Curtis (1995) | FWR, ANAD |  |
|  | 3.00 | 1.00±ND | | 0.03‡ | Hicks and Threlfall (1973) | ND |  |
|  | 15.77 | ND | | ND | Isinguzo (2009) | ANAD |  |
| *Diplostomum* spp*.* | 31.70 | ND | | ND | Bouillon and Curtis (1987) | FWR, ANAD | No |
|  | 23.57 | 46.56±ND | | 42.04 | Due and Curtis (1995) | FWR, ANAD |  |
| *Diplostomum spathaceum* | 6.00 | 2.00±ND | | 0.12‡ | Hicks and Threlfall (1973) | ND | No |
| *Hemiurus levinseni* | 100.00 | 2653.00±2100.80 | | 2653.00‡ | Desdevises et al. (1998) | ANAD | Yes |
| *Lecithaster gibbosus*  *(****Alloopistholecithum gibbosum****)* | 3.00 | 2.00±ND | | 0.06‡ | Bouillon and Dempson (1989) | ANAD | Yes |
|  | 89.25 | 23.60±28.20 | | 20.96‡ | Desdevises et al. (1998) | ANAD |  |
|  | 6.31 | 240.83±337.38 | | 14.49 | Dick and Belosevic (1981) | ANAD |  |
|  | 14.44 | 13.64±ND | | 10.21 | Due and Curtis (1995) | FWR, ANAD |  |
| *Neascus* spp*.* | 13.64 | 1.04±ND | | 0.40 | Due and Curtis (1995) | FWR, ANAD | No |
| *Phyllodistomum umblae* | 2.50 | 17.50±ND | | 0.44‡ | Bouillon and Dempson (1989)^†^ | FWR | No |
|  | 21.60 | 13.00±12.70 | | 2.81‡ | Desdevises et al. (1998) | ANAD |  |
|  | 3.00 | 5.00±ND | | 0.15‡ | Hicks and Threlfall (1973)^†^ | ND |  |
|  | 55.48 | 2.59±ND | | 1.44 | Kolasa and Curtis (1995) | FWR |  |
| *Prosorhynchus squamatus* | 55.53 | 46.29±62.96 | | 49.15 | Dick and Belosevic (1981) | ANAD | Yes |
|  | 3.16 | ND | | ND | Isinguzo (2009) | ANAD |  |
| **Bacteria** | | | | | | | |
| *Aeromonas salmonicida* | 2.08 | ND | | ND | Chapman (2020) | ANAD | No |
| *Candidatus*  *Branchiomonas cysticola* | 85.42 | ND | | ND | Chapman (2020) | ANAD | No |
| *Flavobacterium psychrophilum* | 6.25 | ND | | ND | Chapman (2020) | ANAD | No |
| Gill chlamydia | 4.17 | ND | | ND | Chapman (2020) | ANAD | No |

**Table S3.**  Parasite prevalence (% of infected hosts, with 95% confidence intervals) of anadromous Arctic char (*Salvelinus alpinus*) sampled across five Nunavut areas (Cambridge Bay, Rankin Inlet, Naujaat, Sanirajak, and Sanikiluaq) between 2020 and 2024. Parasites are grouped by major taxonomic class (Trematoda, Cestoda, Nematoda, Acanthocephala, and Copepoda). Bootstrap ANOVA was used to test for differences in prevalence across sampling areas (P-values shown in far-right column). “na” indicates no detection of the parasite in that sampling area. Parasites were extracted from the body cavity (bc), stomach (s), foregut (fg), or hindgut (hg). In some hosts, the gills (g), heart (h), and swim bladder (sb) were also inspected for parasites. Parasites were also collected from the gall bladder (gb), liver (l), gonads (gn), and spleen (sp), but these organs were not sampled in all individuals. The primary site of infection is indicated in bold, and the organs that were not included in the analysis are indicated in italics. Parasites marked with an asterisk identify parasites that were observed in organs that were not sampled in all hosts (i.e., smaller host sample size).

**Table S4**. Mean intensity of metazoan parasites of of anadromous Arctic char (*Salvelinus alpinus*) sampled across five Nunavut areas (Cambridge Bay, Rankin Inlet, Naujaat, Sanirajak, and Sanikiluaq) between 2020 and 2024. Parasites are grouped by major taxonomic class (Trematoda, Cestoda, Nematoda, Acanthocephala, and Copepoda). “na” indicates no detection of the parasite in that sampling area. Parasites were extracted from the body cavity (bc), stomach (s), foregut (fg), or hindgut (hg). In some hosts, the gills (g), heart (h), and swim bladder (sb) were also inspected for parasites. Parasites were also collected from the gall bladder (gb), liver (l), gonads (gn), and spleen (sp), but these organs were not sampled in all individuals. The primary site of infection is indicated in bold, and the organs that were not included in the analysis are indicated in italics. Mean intensity is given with *BC*_a_ (bias-corrected and accelerated) bootstrap 95% confidence intervals, and mean intensity was compared among host populations by bootstrap ANOVA. Parasites marked with an asterisk identify parasites that were observed in organs that were not sampled in all hosts (i.e., smaller host sample size).

**Table S5.** Parasite aggregation of metazoan parasites of anadromous Arctic char (*Salvelinus alpinus*) sampled across five Nunavut areas (Cambridge Bay, Rankin Inlet, Naujaat, Sanirajak, and Sanikiluaq) between 2020 and 2024 quantified using Poulin’s D. Parasites are grouped by major taxonomic class (Trematoda, Cestoda, Nematoda, Acanthocephala, and Copepoda). “na” indicates no detection of the parasite in that sampling area. Parasites were extracted from the body cavity (bc), stomach (s), foregut (fg), or hindgut (hg). In some hosts, the gills (g), heart (h), and swim bladder (sb) were also inspected for parasites. Parasites were also collected from the gall bladder (gb), liver (l), gonads (gn), and spleen (sp), but these organs were not sampled in all individuals. The primary site of infection is indicated in bold, and the organs that were not included in the analysis are indicated in italics. 95% confidence intervals are bias-corrected and accelerated bootstrap intervals (1000 replications). Parasites are often aggregated in their hosts. Parasite aggregation is the “extent to which parasites use the available hosts unevenly, leaving most uninfected while crowding in a few.” D = 0, parasites are uniformly distributed in the hosts. D = 1, all parasites are in a single host (maximum aggregation). Parasites marked with an asterisk identify parasites that were observed in organs that were not sampled in all hosts (i.e., smaller host sample size).

**Table S6.** Comparison of prevalence estimates for parasite species shared between the historical literature and the contemporary empirical assessment of Arctic char (*Salvelinus alpinus*). Historical values represent the range of published prevalence estimates identified in the literature review. Empirical pooled prevalence represents prevalence across all sampled area combined, whereas empirical area range represents the minimum–maximum prevalence observed among contemporary sampling areas. Values in brackets indicate the number of contemporary sampling areas in which each parasite species was detected. Interpretations are qualitative and should be viewed cautiously given geographic and methodological heterogeneity among studies.

**REFERENCES**

Andrews CW, Lear E (1956). The biology of Arctic char (*Salvelinus alpinus* L.) in northern Labrador. J. Fish. Res. Board Can. 13: 843-860.

Bérubé M, Curtis MA (1986). Transmission of *Diphyllobothrium ditremum* to Arctic char (*Salvelinus alpinus*) in two subarctic Quebec lakes. Can. J. Fish. Aquat. Sci. 43(8): 1626-1634.

Beverley-Burton M (1978). Metazoan parasites of Arctic char (*Salvelinus alpinus* L.) in a high Arctic, landlocked lake in Canada. Can. J. Zool. 56(2): 365-368.

Black GA, Lankester MW (1981). The transmission, life span, and population biology of *Cystidicola cristivomeri* White, 1941 (Nematoda: Habronematoidea) in char, *Salvelinus* spp. Can. J. Zool. 59: 2338-2345.

Black GA (1983a). Origin, distribution, and postglacial dispersal of a swimbladder nematode, *Cystidicola stigmatura*. Can. J. Fish. Aquat. Sci. 40: 1243-1248.

Black GA (1983b). *Cystidicola farionis* (Nematoda) as an indicator of lake trout (*Salvelinus namaycush*) of Bering ancestry. Can. J. Fish. Aquat. Sci. 40: 1522-1527.

Black GA (1985). Reproductive output and population biology of *Cystidicola stigmatura* (Leidy) in Arctic char, *Salvelinus alpinus* (L.). Can. J. Zool. 63: 1332-1338.

Bouillon DR, Curtis MA (1987). Diplostomiasis (Trematoda: Strigeidae) in Arctic charr (*Salvelinus alpinus*) from Charr Lake, northern Labrador. J. Wildl. Dis. 23(4): 667-676.

Bouillon DR, Dempson B (1989). Metazoan parasite infections in landlocked and anadromous Arctic charr (*Salvelinus alpinus* Linnaeus), and their use as indicators of movement to sea in young anadromous charr. Can. J. Zool. 67: 2478-2485.

Chapman JM (2020). Factors influencing infectious agent communities and infection burden in free-ranging migratory adult salmonids across Canada. Carleton Univ.

Curtis MA (1983). Parasitism of Arctic char by *Diphyllobothrium ditremum* and *D. dendriticum* in relation to the food web structures of northern lakes. Proc. Second ISACF Workshop Arctic Char.

Curtis MA, Bérubé M, Stenzel A (1995). Parasitological evidence for specialized foraging behavior in lake-resident Arctic char (*Salvelinus alpinus*). Can. J. Fish. Aquat. Sci. 52: 213-221.

Curtis MA, Jensen D (1992). Parasitological sampling of Arctic charr *Salvelinus alpinus* from the vicinity of Igloolik, N.W.T., 1992. Res. Arctic Biol.

Desdevises Y, Arthur JR, Pellerin-Massicotte J (1998). Parasites of anadromous Arctic char (*Salvelinus alpinus* L.) from two sites in Ungava Bay (Quebec, Canada). J. Helminthol. Soc. Wash. 65: 87-93.

Dick TA (1984). Parasites and Arctic charr management: an academic curiosity or practical reality? In: Biology of the Arctic charr.

Dick TA, Belosevic M (1981). Parasites of Arctic charr *Salvelinus alpinus* (Linnaeus) and their use in separating sea-run and non-migrating charr. J. Fish Biol. 18: 547-554.

Due TT, Curtis MA (1995). Parasites of freshwater resident and anadromous Arctic charr (*Salvelinus alpinus*) in Greenland. J. Fish Biol. 46: 759-771.

Eddy SB, Lankester MW (1978). Feeding and migratory habits of Arctic char, *Salvelinus alpinus*, indicated by the presence of the swimbladder nematode *Cystidicola cristivomeri* White. J. Fish. Res. Board Can. 35: 1292-1295.

Gallagher CP, Dick TA, Babaluk JA, Reist JD (2009). Parasite community of Arctic char, *Salvelinus alpinus*, from Lake Hazen and Craig Lake, Quttinirpaaq National Park, Nunavut. Fish. Oceans Can.

Gallagher CP, Dick TA (2010). Trophic structure of a landlocked Arctic char *Salvelinus alpinus* population from southern Baffin Island, Canada. Ecol. Freshw. Fish 19(3): 407-419.

Hanek G, Molnar K (1974). Parasites of freshwater and anadromous fishes from Matamek River System, Quebec. J. Fish. Res. Board Can. 31: 231-234.

Hicks FJ, Threlfall W (1973). Metazoan parasites of salmonids and coregonids from coastal Labrador. J. Fish Biol. 5: 399-410.

Isinguzo IC (2009). The feeding habits of anadromous Arctic char (*Salvelinus alpinus* L.) in Frobisher Bay, Baffin Island in the Eastern Canadian Arctic: diet, parasites and stable isotopes. Univ. Manitoba. Master Thesis.

Jalenques M, Sanders J, Tran L, Beaupré L, Kent M, Lair S (2021). Muscular microsporidian infection in Arctic char *Salvelinus alpinus* from two lakes in Nunavik, Quebec, Canada. Dis. Aquat. Organ. 145: 39-48.

Jamieson JL (1972). Parasites of *Salvelinus alpinus* (Salmonidae) in the northern Foxe Basin, Northwest Territories, with emphasis on those of medical importance. Univ. Toronto. Masters Thesis.

Kolasa KJ, Curtis MA (1995). Seasonal dynamics of helminth parasites in Arctic charr *Salvelinus alpinus* (L.) from a lake resident population in northern Quebec, Canada. Nord. J. Freshw. Res. 71: 90-99.

Laird M (1961). Parasites from northern Canada: II. Haematozoa of fishes. Can. J. Zool. 39: 123-136.

Merks H, Gomes R, Zhu SW, Meymandy M, Reiling SJ, Bolduc S, Mainguy J, Dixon BR (2024). *Toxoplasma gondii* DNA in tissues of anadromous Arctic char, *Salvelinus alpinus*, collected from Nunavik, Québec, Canada. Zoonoses Public Health 71(2): 210-218.

Pufall EL, Jones-Bitton A, McEwen SA, Brown TM, Edge VL, Rokicki J, Karpiej K, Peregrine AS, Simard M (2012). Prevalence of zoonotic anisakid nematodes in Inuit-harvested fish and mammals from the eastern Canadian Arctic. Foodborne Pathog. Dis. 9(11): 1002-1009.

Thomson JA (1957). On the biology of the Arctic char *Salvelinus alpinus* (L.) of Nettilling Lake, Baffin Island, N.W.T. McGill Univ. Masters Thesis.
